# Predictors of perceived success in quitting smoking by vaping: A machine learning approach

**DOI:** 10.1371/journal.pone.0262407

**Published:** 2022-01-14

**Authors:** Rui Fu, Robert Schwartz, Nicholas Mitsakakis, Lori M. Diemert, Shawn O’Connor, Joanna E. Cohen

**Affiliations:** 1 Ontario Tobacco Research Unit, Dalla Lana School of Public Health, University of Toronto, Toronto, Ontario, Canada; 2 Dalla Lana School of Public Health, University of Toronto, Toronto, Ontario, Canada; 3 Children’s Hospital of Eastern Ontario Research Institute, Ottawa, Ontario, Canada; 4 Johns Hopkins Bloomberg School of Public Health, Baltimore, Maryland, United States of America; King Saud University, SAUDI ARABIA

## Abstract

Prior research has suggested that a set of unique characteristics may be associated with adult cigarette smokers who are able to quit smoking using e-cigarettes (vaping). In this cross-sectional study, we aimed to identify and rank the importance of these characteristics using machine learning. During July and August 2019, an online survey was administered to a convenience sample of 889 adult smokers (age ≥ 20) in Ontario, Canada who tried vaping to quit smoking in the past 12 months. Fifty-one person-level characteristics, including a Vaping Experiences Score, were assessed in a gradient boosting machine model to classify the status of perceived success in vaping-assisted smoking cessation. This model was trained using cross-validation and tested using the receiver operating characteristic (ROC) curve. The top five most important predictors were identified using a score between 0% and 100% that represented the relative importance of each variable in model training. About 20% of participants (N = 174, 19.6%) reported success in vaping-assisted smoking cessation. The model achieved relatively high performance with an area under the ROC curve of 0.865 and classification accuracy of 0.831 (95% CI [confidence interval] 0.780 to 0.874). The top five most important predictors of perceived success in vaping-assisted smoking cessation were more positive experiences measured by the Vaping Experiences Score (100%), less previously failed quit attempts by vaping (39.0%), younger age (21.9%), having vaped 100 times (16.8%), and vaping shortly after waking up (15.8%). Our findings provide strong statistical evidence that shows better vaping experiences are associated with greater perceived success in smoking cessation by vaping. Furthermore, our study confirmed the strength of machine learning techniques in vaping-related outcomes research based on observational data.

## 1 Introduction

According to the most recently updated Cochrane systematic review comprising 26 randomized controlled trial studies and 24 non-randomized studies, there is some evidence that nicotine-containing e-cigarettes (vaping) might improve the rate of 6-month smoking abstinence without causing serious adverse events when compared to nicotine replacement therapy (such as nicotine patches) among adult smokers [1]. The effectiveness of nicotine-containing vaping over behavioral support alone is less certain, although overall the evidence appears to be favorable [1, 2].

As vaping might represent a promising option to help some adult smokers quit smoking, it is crucial to identify the characteristics of these smokers to guide the design of health programs that incorporate vaping as a cessation method. Real-world studies have revealed successful vaping-assisted quitting attempters to share a set of unique characteristics, including increased vaping intensity [3], daily use of e-cigarettes [4, 5], longer vaping history [6], the use of a specific type of vaping device [7], and being male [8]. Both qualitative and quantitative studies also point to the use of certain non-tobacco flavors [9, 10] and more positive overall experiences during a vaping-assisted quit attempt [11–17] to be associated with potential cessation benefits.

As most previous studies used regression to identify characteristics of a successful quitter who used vaping, their results are subject to limitations. In the presence of a large pool of variables, all of which are theoretical plausible predictors of smoking cessation, it is often difficult to decide which variables are to be entered into a regression equation and in what mathematical form. Additionally, as variables that describe a successful quitter are likely correlated in intricate manners, completely reflecting these relationships in a regression equation can be challenging. These issues could raise the complexity in a regression analysis and cause concerns of multicollinearity, overfitting and other statistical issues that limit the study findings.

We aim to address these limitations in the current analysis using machine learning, a group of computationally-intensive and data-driven analytical methods that has gained increasing popularity in health research [18–20], including in the research of smoking cessation [21–23] and behaviours of vaping [24, 25]. Compared to conventional regression, machine learning has strengths in mitigating the risk of model overfitting and producing highly accurate and robust prediction [18]. To the best of our knowledge, this is the first application of machine learning in vaping-assisted smoking cessation research. Using survey data collected from a group of adults who tried vaping to quit smoking, we aim to identify and rank the importance of the top five predictors of having perceived success in vaping-assisted smoking cessation.

## 2 Materials and methods

### 2.1 Study design and population

This is a cross-sectional study based in Ontario, Canada. During July and August 2019, we sent an email invitation to 8,109 adult current smokers or recent quitters who had accessed two provincially based smoking cessation initiatives to respond to a survey about vaping to quit smoking, including 4,665 registrants of the Smokers’ Panel, an ongoing initiative that recruits and follows up with smokers to collect data in surveys [26] and 3,444 participants of the Leave the Pack Behind program which provided free nicotine patches, gums and online resources to young adults. We received 1,721 replies (response rate = 21.2%). The Vape to Quit survey (see the complete survey instruments in the S1 File) was administered to individuals who identified themselves to be vaping-assisted cigarette quitters during the past 12 months, i.e., those who reported making at least one serious attempt to quit cigarette smoking (consciously trying to stay off cigarettes for good) by vaping in the past 12 months. Hence, among the 1,721 individuals, we excluded 135 people who did not consent to participate in this study, 219 people who did not smoke cigarettes at all in the past 12 months, 123 people who did not make any serious attempts to quit smoking in the past 12 months, 231 people who did not use e-cigarettes for smoking cessation in the past 12 months, and 20 people who did not give consent for us to use their data from the Vape to Quit Survey (see S1 Fig for a flow chart showing the study sample). These exclusions yielded 993 individuals who met eligibility criteria and thus were administered the survey. All 993 participants completed the survey and received a $10 e-gift card while entering into a draw to win one of two $250 Visa gift cards.

In this study, we further excluded 25 people who did not confirm the use of e-cigarettes for smoking cessation in the survey, one person who did not report smoking cessation outcomes, and 78 people who had no response for at least one entire module of the survey. These exclusions yielded 889 participants in the study sample.

### 2.2 Ethics approval and informed consent

This study received ethical approval by the University of Toronto Health Sciences Research Ethics Board on August 7, 2018 (#10321). Individuals provided written informed consent to participate in this study in addition to allowing us to use their responses to the Vape to Quit Survey in the analysis. Those who did not give consent were excluded from this study.

### 2.3 Outcome

A binary outcome variable was created to represent the status of self-reported success in vaping-assisted smoking cessation in the past 12 months [11]. This variable was measured using the survey question, “Over the past 12 months, would you say that vaping has helped you …?” Participants with perceived success in quitting smoking by vaping answered, “completely helped me quit smoking cigarettes.” Participants with perceived failure answered, “be not at all successful at cutting down smoking cigarettes”, “be somewhat successful at cutting down smoking cigarettes”, or “be very successful at cutting down smoking cigarettes”.

### 2.4 Candidate predictors

Fifty-one characteristics representing a wide spectrum of individual level status and experiences were extracted from the survey to be candidate predictors of perceived success in vaping-assisted smoking cessation. These variables captured sociodemographic information, health status, history of cigarette smoking and quitting, preferences for vaping, side effects from using vaping to quit smoking, the use of other methods while vaping to quit smoking, history of vaping, and substance use. A Vaping Experiences Score (VES) was also used to predict self-reported vaping-assisted smoking cessation. This score was derived from results of a factor analysis on 42 vaping experiences items that we list in S1 Appendix. For each item, participants rated how true the statement was for them on a 7-point scale with 1 being “not true at all” and 7 being “extremely true”. The factor analysis revealed six vaping experiences factors, including Relationships, Flexibility of Vaping, Side Effects, Vaping Devices, Public Reactions and Sensory Functions (see S1 Appendix and the factor analysis done by our group [11]). The VES was estimated as the weighted sum of factor scores over the six factors by the proportion of variance explained. To facilitate interpretation, we categorized individual’s vaping experiences into four levels, based on the four quartiles of the VES: poor (VES≤the first quartile), fair (the first quartile <VES≤the median), good (the median<VES≤the third quartile), and excellent (>the third quartile) [27]. All candidate predictors were represented using a categorical variable.

### 2.5 Statistical analysis

#### 2.5.1 Descriptive statistics and data imputation

Characteristics of successful vaping-assisted quitters vs. unsuccessful vaping-assisted quitters were summarized and compared using the Chi-square test. Overall, less than 0.8% of data were missing, except for two variables—preferred nicotine strength used in vaping (n = 119, 13.4%) and motivation level to quit smoking (n = 80, 9.0%). We used the R package “mice” [28] to generate five imputed copies of our data based on the algorithm of multiple imputation by chained equations, after visual inspection confirmed the assumption that data were missing at random (S2 Appendix). We used the first copy in the primary analysis and returned to the remaining four copies in a sensitivity analysis.

#### 2.5.2 Variable selection

Early literature [29] recommended variable selection procedures prior to machine learning to potentially reduce the risk of model overfitting, prevent spurious associations, and to optimize model training efficiency. The utility of performing variable selection has been confirmed in more recent machine learning applications in health [21, 22, 30], including in the prediction of smoking cessation outcomes [21]. Hence, we implemented two criteria where a predictor was selected into the model if (i) it was one of the six sociodemographic variables or (ii) it was deemed significant in a logistic regression penalized by Lasso (least absolute shrinkage and selection operator) [31]. In criterion (ii), we entered all variables into a logistic regression to be independent predictors of the odds of successful vaping-assisted smoking cessation. A loss function comprising the negative log-likelihood function and the sum of the absolute value of all coefficients was minimized, subject to an unknown tuning parameter that controlled the severity of penalty placed onto the total magnitude of coefficients. This procedure shrank the coefficient of unimportant variables to zero and thereby achieving variable selection. To identify an optimal value of the tuning parameter, a ten-fold cross-validation procedure was performed using the R package “glmnet” [32] such that the cross-validated negative log-likelihood function was minimized (S3 Appendix). Using the optimal value of the tuning parameter, variables with a non-zero coefficient were selected into the analysis by criterion (ii).

#### 2.5.3 Developing and validating a gradient boosting machine

This was a predictive modeling study where we aimed to develop a classification model to predict the status of successful vaping-assisted quitters. Thus, our primary goal was to maximize the predictive power of the final model, rather than to convey any underlying relational or causal relationships as seen in an explanatory modeling study [33]. Interested readers are referred to a previous work done by our group where multivariable logistic modeling was applied to quantify the association between the odds of successful vaping-assisted smoking cessation and various individual-level characteristics [11]. In the present study, we used the R package “gbm” [34] to develop a gradient boosting machine (GBM) model that classified successful vaping-assisted quitters and their unsuccessful counterparts using variables selected from the previous procedure. The GBM is an ensemble model where many weak classification tree models are converted into one single strong model to produce prediction [35]. These tree models are developed sequentially such that each additional tree corrects the prediction error of the preceding tree, and thus continuously updating and improving the prediction. We outlined the analytical procedures below and reserved details in S4 Appendix.

A ratio of 7:3 was used to split the data randomly into a larger set used for model training (n = 623) and a smaller set reserved for model testing (n = 266). Because the number of successful vaping-assisted quitters is substantially less than that of unsuccessful quitters, with a ratio of 1:4, we applied SMOTE (synthetic minority over-sampling technique) [36] in the R package “DMwR” [37] to create synthetic samples of successful quitters on the basis of nearest neighbor to achieve a more balanced training set. Using data from the resampled training set, 5000 tree models were developed sequentially using a gradient descent method (assuming a learning rate of 0.01) to classify smoking cessation status, assuming each tree had at most 6 binary splits and at least 5 observations in its terminal nodes. Extensive tuning procedures were conducted using an internal ten-fold cross-validation process to prune tree models and to identify optimal values for the other parameters of GBM, in order to rule out the possibility of model overfitting while maintaining the overall performance of the GBM.

We used the area under the receiver operating characteristic (ROC) curve, known as the AUC, to measure performance of the GBM on the testing set. The AUC ranges from 0.5 to 1 with higher values indicating greater discriminatory ability. A model with an AUC ≥ 0.80 is generally considered to be strong [38].

#### 2.5.4 Ranking of variable importance

We calculated a score for each predictor to represent its relative importance in the training of the GBM model using the “relative.influence” function [34] in the “gbm” package. To calculate the score, for each variable, we computed the improvement in the Gini Index at each split in each tree, and then calculated the average improvements across all trees in the GBM. We scaled this score to be between 0%-100% such that the variable with the highest score had 100% importance [35]. The top five predictors were identified and ranked using a bar plot.

#### 2.5.5 Visualizing partial dependences

We used the “plot.gbm” function [34] in the “gbm” package to examine graphically the partial dependence of the predicted probability of reporting success in vaping-assisted smoking cessation based on the GBM model on values of the five top predictors. This procedure, recommended to be performed after the ranking of variable importance [39], provides visual interpretations on the approximated relationship between the top predictors and the outcome while other variables are “integrated out” [35]. The partial dependence was estimated for each of the top five predictor at each of its value as the marginal success rate predicted by the GBM model by forcing all data to have the same value for this predictor [35]. Visualizations were performed using the “ggplot2” package [40].

#### 2.5.6 Sensitivity analysis

We first repeated all analytical procedures on each of the four remaining imputed datasets to identify the effect of the missing values. We then assessed a parsimonious GBM model that was trained using only the top five predictors [41]. After that, we deliberately omitted the VES variable from the model to assess its impact on the testing results. And finally, we estimated a conventional multivariable logistic model with data from the original training set by entering all variables as independent predictors of vaping-assisted cigarette cessation. Performance of the logistic model was assessed on the same testing set. Analyses were performed on R version 3.6.1 (R Foundation for Statistical Computing).

## 3 Results

### 3.1 Characteristics of the study sample

Of the 889 participants, 174 (19.6%) reported to have successfully quitted smoking by vaping in the past 12 months (Table 1). Compared to their unsuccessful counterparts, these successful quitters were less likely to be female (49.1% vs. 58.0%) but were more likely to be employed or self-employed (82.8% vs. 75.1%). Self-reported health status did not differ between the two groups, although successful quitters were more likely to report having no formally diagnosed health conditions (38.5% vs. 29.7%). Furthermore, successful quitters had higher motivation to quit smoking in general (80.5% vs. 63.4%), but had lower likelihood of having set up a quit date in advance (33.3% vs. 45.0%).

**Table 1:** Comparing characteristics of participants by perceived success in vaping-assisted smoking cessation.

Characteristics	Unsuccessful quitters (n = 715)		Successful quitters (n = 174)		p ^a^
**Sociodemographic factors**	**n**	**%**	**n**	**%**	
**Age**					0.30
In their 20s	220	31.4	58	33.9	
In their 30s	239	34.1	59	34.5	
In their 40s	89	12.7	27	15.8	
50 or above	153	21.8	27	15.8	
**Female**	413	58.0	85	49.1	0.04
**Non-White race**	144	20.3	24	13.8	0.06
**Highest education**					0.85
High school or below	216	30.4	54	31.0	
College diploma	267	37.6	68	39.1	
University or above	228	32.1	52	29.9	
**Employed or self-employed**	534	75.1	144	82.8	0.04
**Marital status**					0.11
Single, never married	307	43.2	62	35.6	
Married or living with a partner	329	46.3	96	55.2	
Divorced, separated, widowed	75	10.5	16	9.2	
**Health status**	**n**	**%**	**n**	**%**	
**Physical health status**					0.16
Excellent or very good	166	23.2	29	16.7	
Good	285	39.9	73	42.0	
Fair or poor	263	36.8	72	41.4	
**Mental health status**					0.63
Excellent or very good	199	27.9	43	24.7	
Good	244	34.2	59	33.9	
Fair or poor	271	38.0	72	41.4	
**Stress level**					0.24
Not very stressful	302	42.2	65	37.4	
Quite stressful	263	36.8	76	43.7	
Extremely stressful	150	21.0	33	19.0	
**Having been formally diagnosed with**					
Depression	288	40.3	68	39.1	0.84
Anxiety	321	44.9	64	36.8	0.06
Attention deficit hyperactivity disorder	82	11.5	23	13.2	0.61
Asthma	99	13.8	15	8.6	0.09
Chronic pain	97	13.6	13	7.5	0.04
Other conditions ^b^	174	24.3	36	20.7	0.36
Did not have any formally diagnosed health condition	212	29.7	67	38.5	0.03
**History of cigarette smoking and quitting**	**n**	**%**	**n**	**%**	
**Started smoking at age 18 or older**	436	61.8	109	62.6	0.90
**Having tried to quit smoking 6 more times in lifetime**	245	35.0	70	40.9	0.17
**Highly motivated to quit smoking** ^c^	409	63.4	132	80.5	<0.001
**Having set up a quit date**	322	45.0	58	33.3	0.007
**Preferences for vaping**	**n**	**%**	**n**	**%**	
**Using a pod system to vape**	216	30.9	77	44.5	0.001
**Typical nicotine strength in vape**					<0.001
None or <0.5%	137	22.8	66	39.1	
0.5%-2.0%	352	58.6	67	39.6	
2.1% or above	112	18.6	36	21.3	
**Typical flavor in vape**					
Fruity	387	54.1	101	58.0	0.40
Candy or beverage	250	35.0	63	36.2	0.83
Mint or menthol	212	29.7	49	28.2	0.77
Tobacco	302	42.2	59	33.9	0.06
Other flavors	66	9.2	17	9.8	0.94
**Vape at least 10 times daily**	365	51.6	118	69.0	<0.001
**Puffs per vape**					0.04
Less than 5	240	34.0	75	44.1	
5–9	276	39.1	60	35.3	
10 or more	190	26.9	35	20.6	
**Time after waking up to vape**					0.002
Within 15 minutes	239	33.9	67	39.2	
15–60 minutes	234	33.2	71	41.5	
Beyond 1 hour	231	32.8	33	19.3	
**Side effects from vaping**	**n**	**%**	**n**	**%**	
Mouth irritation	136	19.0	34	19.5	0.96
Throat irritation	449	62.8	94	54.0	0.04
Chest irritation	162	22.7	31	17.8	0.20
Headache	193	27.0	36	20.7	0.11
Nausea, vomiting, or lightheaded	249	34.8	58	33.3	0.78
Did not experience any side effect	175	24.5	54	31.0	0.09
**Use of other methods while vaping to quit smoking**	**n**	**%**	**n**	**%**	
Did not use any other method	253	35.4	88	50.6	<0.001
Electronic sources ^d^	119	16.6	19	10.9	0.08
Professional help ^e^	342	47.8	63	36.2	0.007
Family and friend support	164	22.9	39	22.4	0.96
Alternative therapy	94	13.1	14	8.0	0.09
Using other tobacco products	542	75.8	155	89.1	<0.001
**History of vaping**	**n**	**%**	**n**	**%**	
**Having vaped 100 times in lifetime**	361	50.6	129	74.1	<0.001
**Started vaping to quit smoking**	526	73.8	159	91.4	<0.001
**Lifetime attempts to quit smoking by vaping**					<0.001
First time	190	26.6	95	54.6	
Second time	243	34.0	51	29.3	
Third time	126	17.6	19	10.9	
Forth or more	156	21.8	9	5.2	
**Time since the start of last attempt to quit smoking by vaping**					<0.001
Less than a month	166	23.2	11	6.3	
1–12 months	484	67.7	120	69.0	
More than 12 months	65	9.1	43	24.7	
**Current substance use status**	**n**	**%**	**n**	**%**	
**Alcohol**	536	75.6	135	78.5	0.49
**Cannabis**					0.19
Nonusers	318	44.7	76	43.7	
Occasional	185	26.0	56	32.2	
Daily or almost daily	208	29.3	42	24.1	
**Waterpipe**	101	14.3	9	5.3	0.002
**Other tobacco products** ^f^	102	14.5	11	6.5	0.007

^a^ Comparison of characteristics between successful and unsuccessful vaping-assisted smoking quitters was performed using the Chi-square test.

^b^ Other conditions include diabetes, heart disease, lung disease, cancer, alcohol issues and any other health conditions.

^c^ Participants were asked to rate the level of motivation to quit smoking on a scale of 0–100. We defined “highly motivated” individuals to be those scored 75 or above.

^d^ Electronic sources include online sources, quit line, quit apps and text messaging service.

^e^ Professional help includes prescription medications, nicotine replacement therapy, and advise from healthcare professionals.

^f^ Other tobacco products include cigars, little cigars, and heated tobacco.

The two groups of participants showed significant differences in terms of their preference for and history of vaping. Specifically, successful quitters were more likely to use a pod system (44.5% vs. 30.9%), to use 2.1% or higher nicotine in vape (21.3% vs. 18.6%), to vape at least 10 times daily (69.0% vs. 51.6%), to vape within 15 minutes after waking up (39.2% vs. 33.9%), and having vaped 100 times (74.1% vs. 56.0%). When discussing the use of vaping for quitting smoking, successful quitters were more likely to have started vaping specifically for smoking cessation (91.4% vs. 73.8%) and to report less previously failed attempts (first time attemper: 54.6% vs. 26.6%). Regarding their latest quit attempt, successful quitters were more likely to report that they had started this attempt more than 12 months ago (24.7% vs. 9.1%). During this attempt, successful quitters were less likely to report throat irritation (54.0% vs. 62.8%) or the concurrent use of another method besides vaping to aid quitting. In terms of substance use, successful quitters were less likely to be current users of waterpipe (5.3% vs. 14.3%) or other tobacco products such as cigars (6.5% vs. 14.5%).

**Fig 1:**
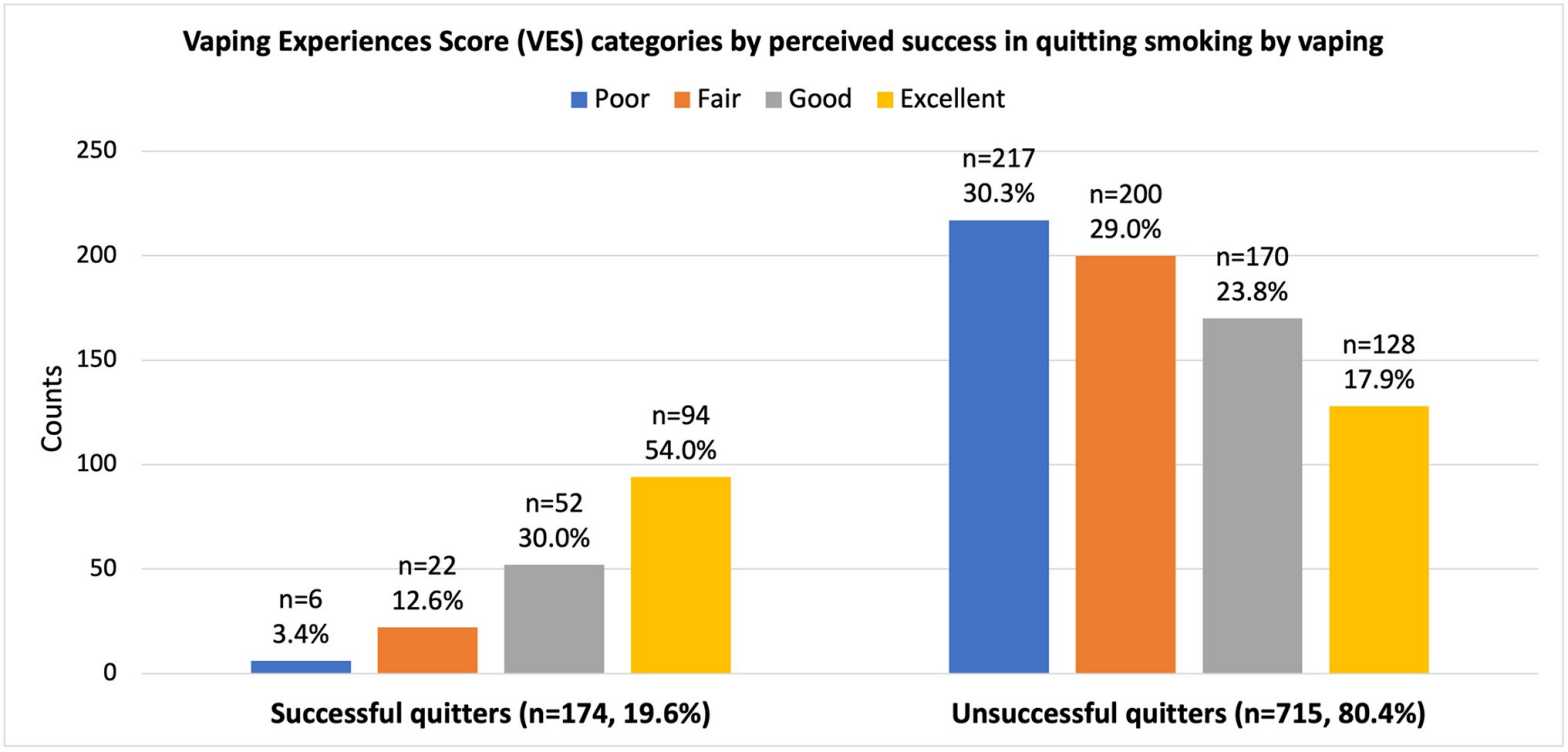
Vaping Experiences Score (VES) categories by perceived success in quitting smoking by vaping.

Fig 1 illustrates the VES categories between the two groups of quitters. In general, successful quitters were significantly more likely to have more positive experiences as measured by the VES (p<0.001). Notably, more than half (54.0%) of successful quitters rated their experiences to be excellent compared to just 17.9% of unsuccessful quitters.

### 3.2 Model structure and performance

**Fig 2:**
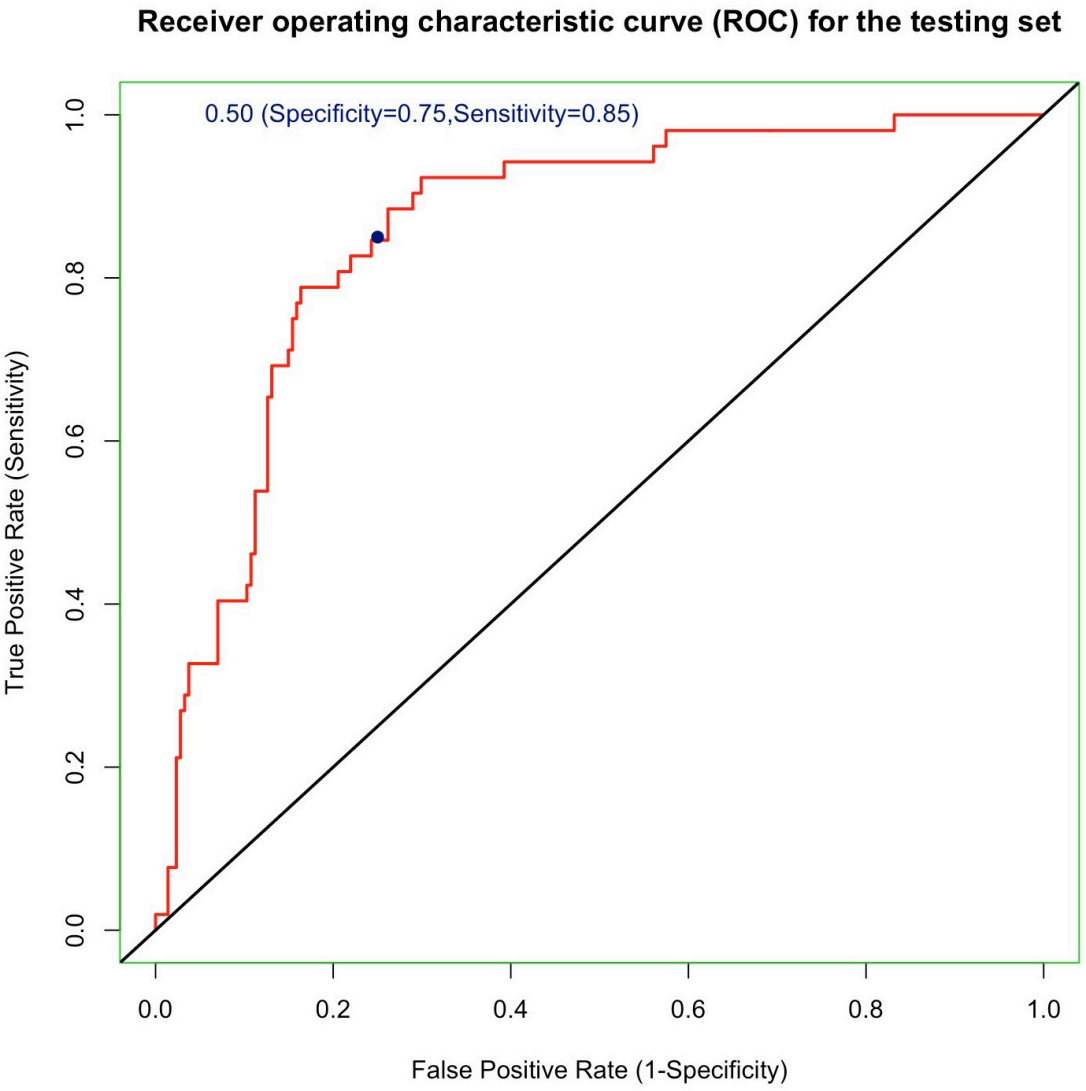
Receiver operating characteristics curve of the gradient boosting machine model on the testing set.

The Lasso logistic regression excluded 9 unimportant variables that were mostly related to preferred flavors of vaping (S3 Appendix). Entering the remaining 42 predictors into the analysis yielded a final GBM model with an AUC of 0.865 (Fig 2), specificity of 0.750 (95% confidence interval [CI] 0.614 to 0.863), sensitivity of 0.851 (95% CI 0.798 to 0.902) and accuracy of 0.831 (95% CI 0.780 to 0.874) on the testing set.

### 3.3 Top five predictors of perceived success in vaping-assisted smoking cessation

**Fig 3:**
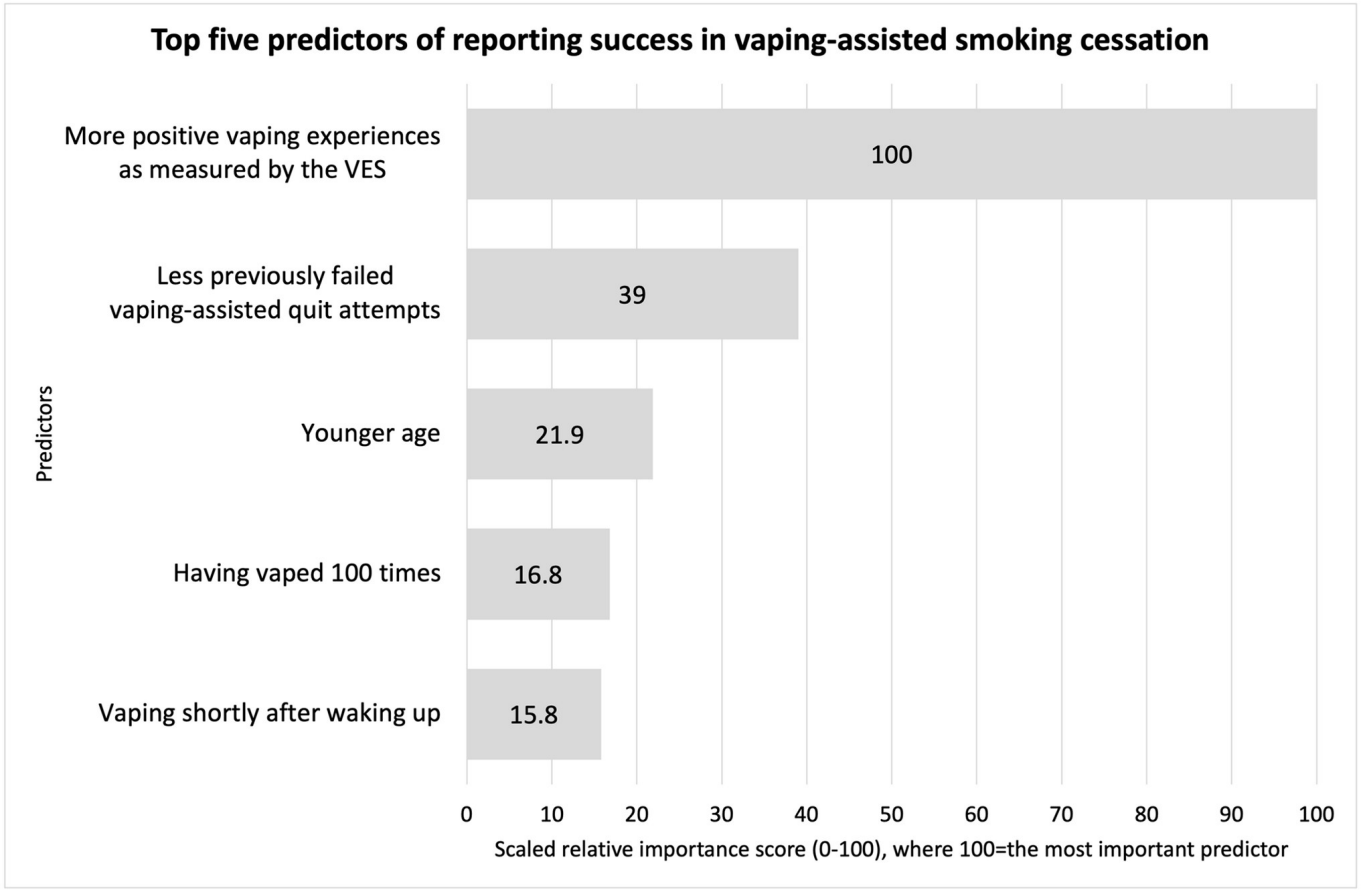
Top five predictors of reporting success in vaping-assisted smoking cessation.

We identified and ranked the importance of the top five predictors of self-reported vaping-assisted smoking cessation on the basis of a score between 0%-100% (Fig 3). These predictors were more positive experiences measured by the VES (100%), less previously failed quit attempts by vaping (39.0%), younger age (21.9%), having vaped 100 times (16.8%) and vaping shortly after waking up (15.8%).

### 3.4 Partial dependence

To enhance the interpretability of the top five predictors, we estimated the partial dependence of the predicted probability of perceived success in vaping-assisted smoking cessation on the values of these predictors (S2 Fig). Having positive vaping experiences alone was found to be strongly associated with a higher rate of successful quitting, as the model predicted the marginal success rate of participants with poor, fair, good, and excellent vaping experiences to be 4.8%, 13.4%, 46.2%, and 77.1%. The model also suggested that, compared with participants who vaped to quit smoking for the first time, those with previous failed attempts were less likely to report success. Notably, the marginal success rate decreased consistently from 68.6% (first attempt) to 20.7% (second attempt) to 10.5% (third attempt) to just 2.5% (four or more attempt).

The model found a decreasing trend of smoking cessation by age, where the youngest age group (20–29) had the highest marginal probability of quitting (49.5%), followed by participants in their 30s (30.8%), in their 40s (19.4%), and those who were 50 years old or above (5.2%). Furthermore, the marginal success rate decreased from 39.7% for participants who had vaped 100 times or more to 19.4% for those who hadn’t vaped 100 times. Finally, the marginal probability of quitting decreased by longer time between waking up and first vaping session. Specifically, smokers who started vaping within 15 minutes after waking up reported the highest marginal success rate (46.9%), which reduced to 20.8% for those who vaped in 15–60 minutes, and to 10.4% for those vaped at least an hour thereafter.

### 3.5 Sensitivity analysis

Results of sensitivity analyses are presented in S5 Appendix. Repeating procedures on each of the remaining four imputed data copies yielded very similar models with AUCs ranging from 0.855 to 0.869. Minor discrepancy emerged as in one iteration, time after waking up to vape (15.9%) replaced having vaped 100 times (14.4%) as the fourth most important predictor. A parsimonious model that was trained using only the top five predictors reached slightly reduced AUC of 0.825, sensitivity of 0.664 (95% CI 0.609 to 0.735), specificity of 0.827 (95% CI 0.704 to 0.924), and accuracy of 0.700 (95% CI 0.636 to 0.750), on the testing set, but did not change the importance ranking for the top five predictors or their importance scores. Excluding the VES variable reduced model performance on the testing set, resulting in an AUC of 0.772, sensitivity of 0.463 (95% CI 0.399 to 0.532), specificity of 0.846 (95% CI 0.724 to 0.931) and accuracy of 0.538 (95% CI 0.476 to 0.599). In this model, the top five predictors were the number of vaping-assisted quit attempts (100%), age (55.7%), having vaped 100 times (51.7%), using a pod system (45.6%) and time after waking up to vape (37.4%). Finally, a multivariable logistic model treating all variables as independent predictors of vaping-assisted cigarette cessation was estimated using the data from the original training set. The results of this analysis corroborated the importance of four of the top five predictors but ruled out the significance of time after waking up to vape. Specifically, when compared to participants with poor vaping experiences, those with fair, good or excellent experiences were associated with 2.818-fold (95% CI 1.616 to 5.075, p<0.001), 7.593-fold (95% CI 2.475 to 33.171, p = 0.002) and 15.660-fold (95% CI 5.671 to 52.801, p<0.001) odds of reporting a successful vaping-assisted smoking cessation. On the testing set, the logistic model reached an AUC of 0.701, sensitivity of 0.940 (95% CI 0.910–0.979), specificity of 0.248 (95% CI 0.147–0.362) and accuracy of 0.808 (95% CI 0.764–0.848).

## 4 Discussion

Using survey data from 889 Canadian smokers who used vaping to quit smoking in the past 12 months, we developed and validated a machine learning model that identified predictors of perceived success in vaping-assisted smoking cessation. The final model achieved high performance and suggested the VES, number of quit attempts by vaping, younger age, having vaped 100 times, and vaping shortly after waking up were the most important predictors of self-reported successful quitting. These results are robust to missing data and alternative model specification.

About 20% (19.6%) of participants in our sample reported success in quitting smoking by vaping, a rate that closely resembles observations from both trial and real-world settings in Europe and the US [1, 13, 42–45]. Thanks to a novel machine learning technique, we were able to build upon results of previous studies and more importantly, to identify and also rank the importance of, variables that were correlated with higher rate of perceived success in vaping-assisted smoking cessation. Compared with conventional logistic regression approach, we showed machine learning greatly improved model performance in the presence of a large number of variables and limited sample size. Hence, we believe that machine learning is well-suited in health outcomes research as it leverages computational power to effectively and directly identify influential variables and to quantify the strength of relationships. In our case, the ranking yielded by the machine learning analysis provides practical and actionable inputs on priority-setting in the development of interventions that maximize the effectiveness of e-cigarettes as a smoking cessation device.

Consistent with early studies done by our group [11, 46], we identified the VES, an index for measuring the experiences of smokers during a vaping-assisted quit attempt, as the most important predictor of self-reported smoking cessation. Notably, when holding other characteristics constant, smokers with excellent vaping experiences were associated with 16-times averaged predicted probability of quitting smoking compared to those who scored in the lowest quartile (77.1% vs. 4.8%). Furthermore, excluding the VES variable resulted in a decrease in model performance, especially in sensitivity (i.e., the ability to correctly identify true quitters). To give a more concrete example, suppose a female first-time quitting attempter in her 40s has vaped 100+ times and is currently vaping within 15 minutes after waking up (while holding other characteristics at reference level). The machine learning model predicts her perceived success rate during the current (first) quit attempt to be 2.8%, 9.1%, 12.4%, and 39.4%, corresponding to having poor, fair, good, and excellent vaping experiences. This implies improving this individual’s vaping experiences from poor to excellent may be associated with a drastic, 14-fold increase in her probability of self-reported smoking cessation. Another point worth noting is that, compared to other top predictors, the vaping experiences of smokers during a quit attempt are relatively modifiable and could be effectively improved by interventions, such as switching to a more adequate vaping device and increased monitoring for side effects [11]. Hence, we believe the paramount importance of vaping experiences yielded by our analysis points to a feasible opportunity to prioritize strategies that enhance the experiences of smokers who rely on vaping as the main cessation aid as a way to potentially improve their likelihood of cessation. However, our results need to be interpreted with caution as they did not infer vaping experiences to be a determinant of successful smoking cessation. Hence, more in-depth research needs to explicate the specific mechanism of vaping experiences in influencing smoking cessation outcomes to support policy actions.

The second and third most important predictors were the number of quit attempts by vaping and the age of smoker, although both were much less important compared to the VES. The probability of quitting decreased consistently with increasing number of quit attempts, where we found first-time quitters were more than 30-fold more likely to quit on average compared to those who had already failed 3+ times (68.6% vs. 2.5%). A similar trend was revealed in age, as the youngest smokers in their 20s were associated with about 8-times the chance of quitting smoking by vaping compared to older smokers aged 50 or above (49.5% vs. 5.2%).

An interesting finding was a potential positive relationship between the likelihood of smoking cessation by vaping and indicators of vaping addiction. The model suggested that both established vapers (defined as having vaped 100 times or more) and those who vaped shortly after waking up were associated with higher success rates with vaping in quitting smoking. These observations imply that individuals with perceived success in vaping-assisted smoking cessation may have simply shifted their mode of nicotine consumption from combustible cigarettes to e-cigarettes, without eliminating the use of nicotine.

Finally, the analysis confirmed the importance of previously identified predictors of smoking cessation, however they were not as important as our top five predictors. For example, daily vaping frequency [3] was identified to be the eighth important predictor with a score of 4.4%. Specifically, those who vaped <10 and 10+ times daily had marginal cessation rate of 26.7% and 28.8%, respectively. Furthermore, vaping device [7] was the sixteenth predictor with a score of 1.8% where using a pod system was associated with slightly higher marginal probability of quitting than using other devices (36.8% vs. 27.5%).

Our analysis has limitations. First, due to the cross-sectional nature of the survey data, we were able to identify correlates only, rather than true predictors, of perceived smoking cessation by vaping. Additionally, we were unable to determine if participants had maintained e-cigarette use after quitting smoking. These limitations should be addressed in longitudinal studies. Second, the cross-sectional data and the data-driven nature of machine learning did not permit us to draw any causal conclusions. Specifically, we were unable to attest if vaping experiences (reflected in the VES) constituted a causal factor of successful vaping-assisted smoking cessation, nor did we conduct any analysis to statistically rule out a reverse association between vaping experiences and improved smoking cessation outcomes. These limitations could be addressed by future researchers who apply a rigorous quasi-experimental method to a longitudinal dataset. Third, we did not have access to a biomedically verified smoking cessation measure and instead, relied on a self-reported quitting status. Additionally, rather than directly asking participants about their vaping-assisted quitting status (i.e., “did you quit smoking specifically using vaping for at least one week after your most recent quit attempt?”), we employed a more subjective measure by asking about their levels of perceived usefulness of using vaping to cut down cigarette smoking. Future studies can overcome this limitation by either including lab procedures to establish the abstinence outcome objectively or enhance the survey design by incorporating a direct and less subjective self-reported quitting measure. Forth, our sample size is small when compared to previous machine learning applications in health [27, 41]. Because the performance of machine learning models depends directly on the volume of data, the limited sample size may have impeded our ability to derive a better model. However, even with this small sample, our model reached an AUC of 0.865, indicating high discriminatory ability [38]. Finally, the use of a convenience sample of adult smokers in Ontario, Canada may have reduced the generalizability of our findings to a broader population. Future studies should revisit this topic with larger representative samples.

Our study provides several implications for future research. As we expand the current evidence on a potential link between the experiences of smokers during a vaping-assisted smoking cessation attempt and their quitting outcome [11–16], more statistical investigations are warranted to understand the direction and the strength of this association. Furthermore, although we showed our results to be largely robust to the small percentage of data (0.8%) that were absent from the dataset, future researchers that encounter more severely missed data might want to adopt a more sophisticated analytical pipeline that incorporates multiple imputations within the machine learning framework to generate robust findings [47, 48]. Next, we showed the gradient boosting machine outperformed the traditional logistic regression in predicting the status of vaping-assisted smoking cessation, which implies the utility of machine learning in devising predictive tools to assist smoking cessation efforts. Future studies need to explore the feasibility of deploying such machine learning-enabled tools in a real-world setting. Finally, with a surging interest in enhancing the transparency and the explainability of black-box machine learning models as well as advancing machine learning methods to gain the capability of causality testing (i.e., causal learning) [49–51], future researchers might consider experimenting with these novel analytical techniques in studying the mechanism of a successful vaping-assisted smoking cessation.

## 5 Conclusions

Using machine learning, we identified five person-level characteristics—including vaping experiences (measured by a Vaping Experiences Score), number of quit attempts by vaping, age, having vaped 100 times and the time after waking up to vape—to be the top five predictors of the probability of perceived success in vaping-assisted smoking cessation. These results provide valuable implications for interventions aim at maximizing the effectiveness of e-cigarettes as a potential cessation device.

## Supporting information

S1 Fig A flow chart showing the inclusion of participants into the study sample. (DOCX)

S2 Fig Partial dependence plots depicting the marginal probabilities of perceived success in vaping-assisted smoking cessation based on the top five predictors. Abbreviations: VES, Vaping Experiences Score.
(DOCX)

S1 Appendix Derivation of the Vaping Experiences Score (VES) using factor analysis. (DOCX)

S2 Appendix Inspection and imputation of missing values. (DOCX)

S3 Appendix Variable selection. (DOCX)

S4 Appendix Train and tune a gradient boosting machine (GBM). (DOCX)

S5 Appendix Procedures and results of sensitivity analysis. (DOCX)

S1 File Vape to Quit Survey instruments. (PDF)
