# Supplementary figures and images for: Predictors of perceived success in quitting smoking by vaping: A machine learning approach
Source: PLoS One. 2022 Jan 14;17(1):e0262407. doi: 10.1371/journal.pone.0262407 (PMC8759658; doi:10.1371/journal.pone.0262407)

**S1 Figure.** A flow chart showing the inclusion of participants into the study sample

**
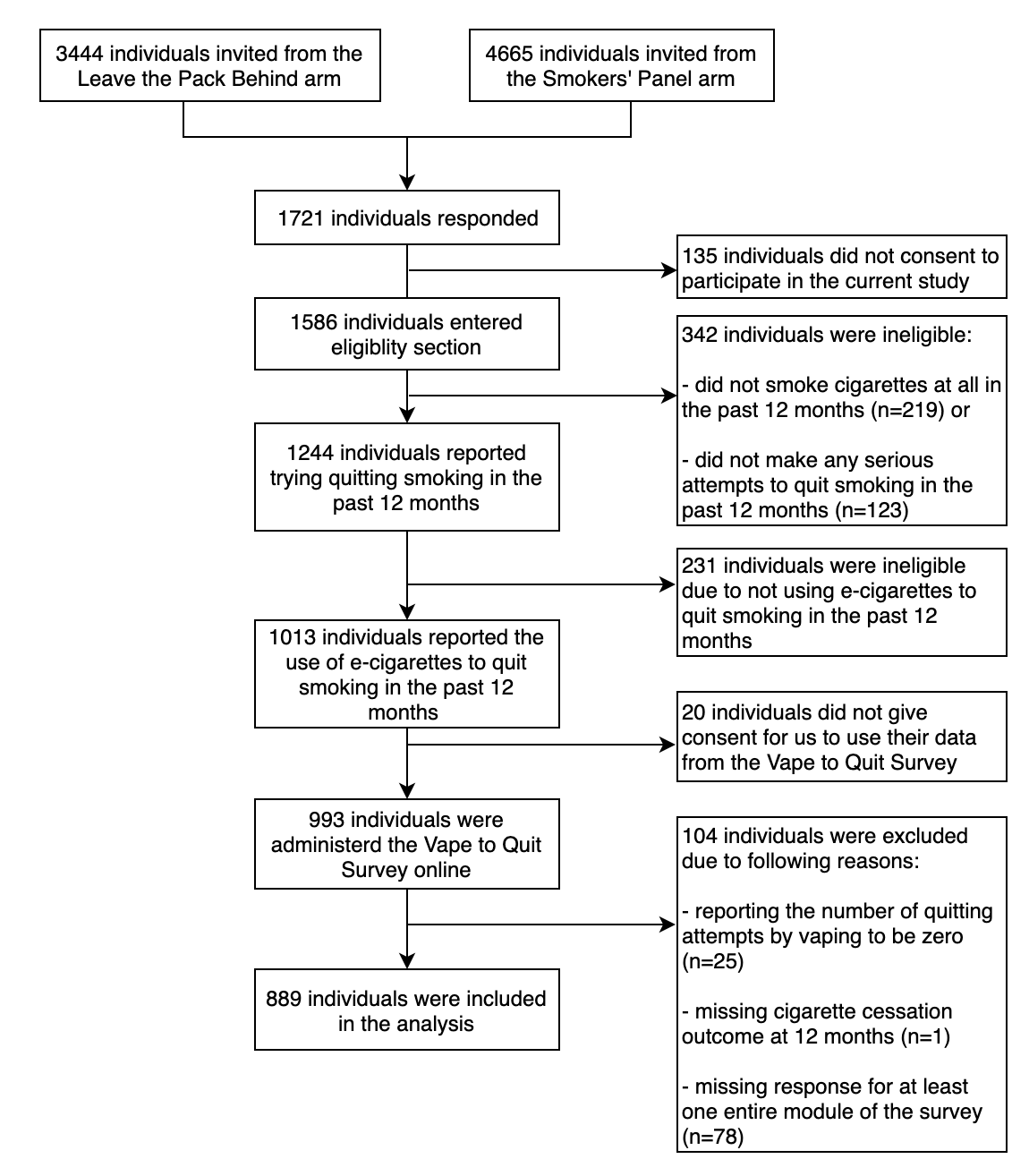
**

Supplement: S1 Fig — (DOCX) [file pone.0262407.s001.docx]
