# Supplementary material for: Predictors of perceived success in quitting smoking by vaping: A machine learning approach
Source: PLoS One. 2022 Jan 14;17(1):e0262407. doi: 10.1371/journal.pone.0262407 (PMC8759658; doi:10.1371/journal.pone.0262407)
Supplement: S2 Fig — Abbreviations: VES, Vaping Experiences Score. (DOCX) [file pone.0262407.s002.docx]

**S2 Fig. Partial dependence plots depicting the marginal probabilities of perceived success in vaping-assisted smoking cessation based on the top five predictors.**


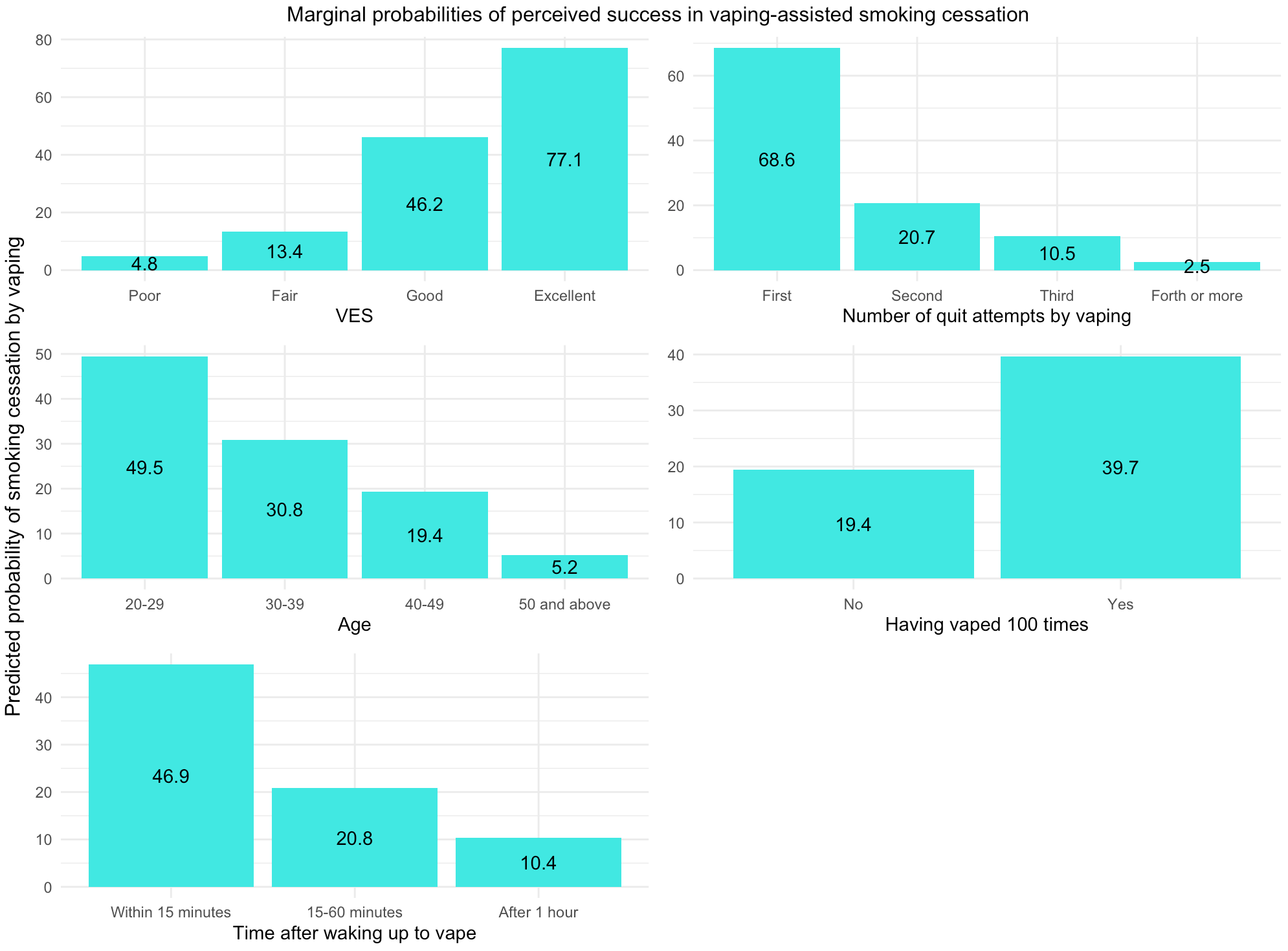


Abbreviations: VES, Vaping Experiences Score.
