# Supplementary material for: Predictors of perceived success in quitting smoking by vaping: A machine learning approach
Source: PLoS One. 2022 Jan 14;17(1):e0262407. doi: 10.1371/journal.pone.0262407 (PMC8759658; doi:10.1371/journal.pone.0262407)
Supplement: S1 Appendix — (DOCX) [file pone.0262407.s003.docx]

**S1 Appendix.** Derivation of the Vaping Experiences Score (VES) using factor analysis

The Vaping Experiences Score (VES) is a proxy measure of smokers’ experiences during a cigarette quitting attempt using vaping. It is derived from an exploratory factor analysis by applying the function “fa” in the R package “psych” to 42 vaping experiences items (listed below). In the survey, for each item, participants rated how true the statement was to them on a 7-point scale with 1 being “not at all true” and 7 being “extremely true”.

| **42 vaping experiences items included in the survey** |
| --- |
| Vaping doesn’t leave a horrible taste in my mouth. |
| I like the number of nicotine strengths available |
| When I vape, I can take as many or as few puffs as I want. With cigarettes I often smoked more than I wanted because I was trying to finish the whole cigarettes |
| With vaping, it is easier to lower my amount of nicotine intake than it was to cut down on the how many cigarettes I used to smoke |
| Vaping is easier than real cigarettes |
| I really like the flavors |
| I can vape almost anywhere |
| Vaping costs less than smoking |
| I like my device because it is portable, durable, and came in nice colours |
| Vaping satisfied the associated sensations related to smoking cigarettes |
| The vape store employees are very knowledgeable and helpful |
| I don’t smell like cigarettes all the time |
| I feel less dirty when vaping |
| By vaping, I don’t have the guilt of smoking cigarettes |
| I feel like I can breathe again |
| I feel better |
| I feel like it is worth taking care of my teeth again |
| Vaping is better for my health than smoking |
| I can smell things again |
| I have more energy |
| My sense of taste returned |
| My family members prefer me vaping because of the health benefits |
| I like that I can tell people I don’t smoke |
| I prefer vaping because I don’t like the stigma of smoking |
| I am more accepted by my non-smoking friends when I vape |
| People think that vapor is the same as second-hand smoke |
| People judge you for vaping |
| I feel stupid using my vape in public |
| People don’t like me vaping around them |
| People often complain about the amount of vapor emitted by the devices and it makes it hard to smoke them in many places |
| It is frustrating to have to vape outside and be around people smoking cigarettes – it makes me just want to smoke |
| I worry about the effects of vaping on my health |
| When I inhale too quickly, I burn my throat which turns into a coughing fit |
| The vapour makes me choke at first and it is hard to breathe |
| Vaping makes me dehydrated |
| I feel like I am replacing one habit with another |
| I feel like I had to constantly vape to feel satisfied |
| The fruit or candy flavors did not satisfy my craving for a cigarette |
| I am unsure about where I can vape indoors |

In order to reduce the dimension of vaping experiences from 42 to just a few important factors, we applied factor analysis and extracted six factors (determined by functions in the R package “nFactors”) using the minimum residual method and varimax rotation. The 6-factor solution accounts for 44% of total variations. We estimated the factor score of participants using the Barlette method – this gave an unbiased estimate of the true score. To generate a global score over the six factors, we calculated the weighted sum using the proportion of variance explained by each factor:

VES = 0.2604 * Relationships score +

0.2405 * Flexibility of Vaping score +

0.1589 * Side Effects score +

0.1185 * Vaping Devices score +

0.1153 * Public Reactions score +

0.1063 * Sensory Functions score

We categorized VES based on the three quantiles: Poor (<=25%), fair (25%-50%), good (50%-75%), and excellent (>75%). In our data, this corresponds to the following categorization rule:

- Poor: VES <= 0.3009
- Fair: 0.3009 < VES <= 0.0132
- Good: 0.0132 < VES <= 0.3303
- Excellent: VES > 0.3303

The figure on the next page depicts the distribution of VES in our sample:


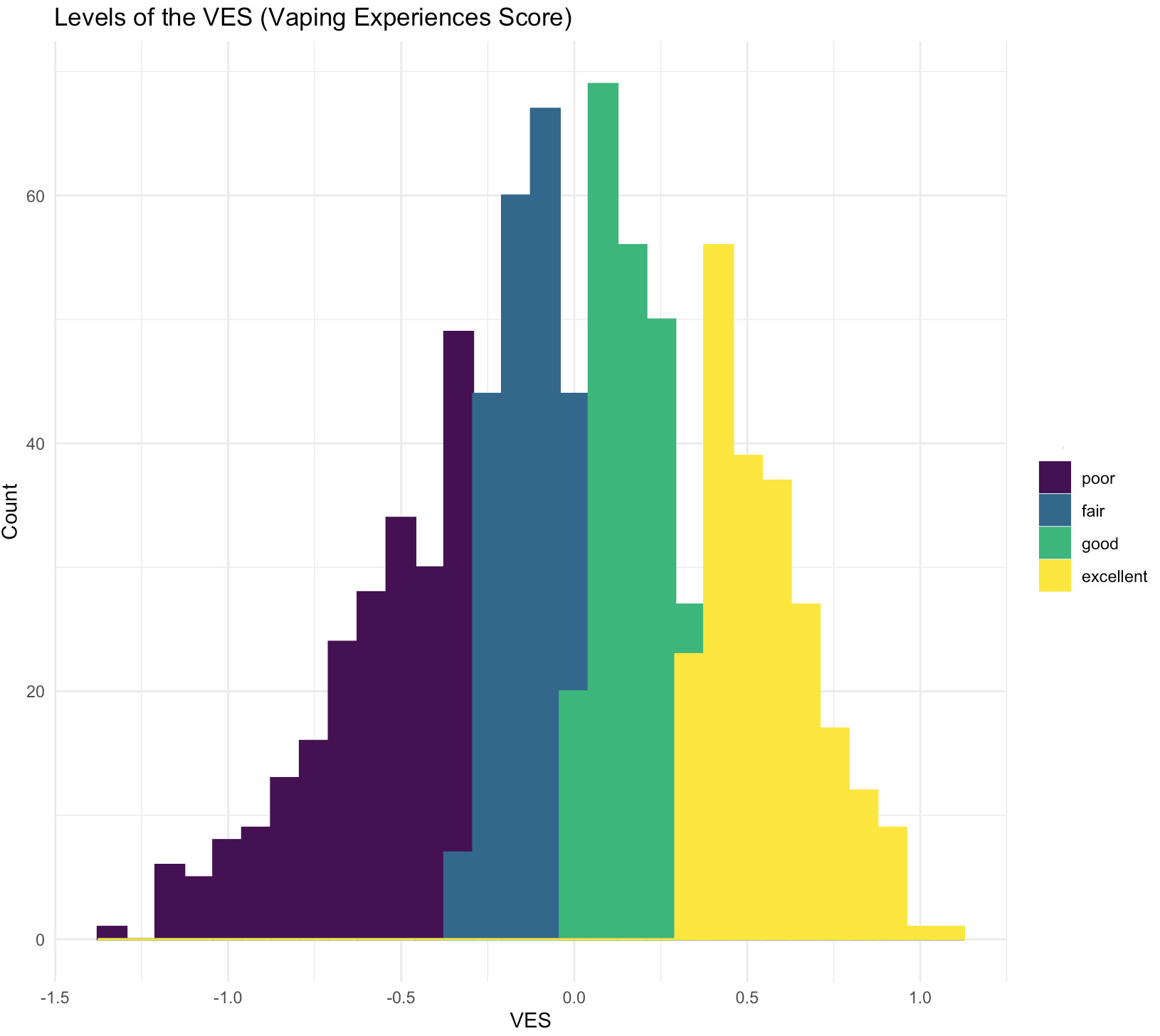


Additional information on the factor analysis can be found in an article published by our group:

Fu R, O’Connor S, Diemert L, Pelletier H, Eissenberg T, Cohen J, Schwartz R. Real-world vaping experiences and smoking cessation among cigarette smoking adults. Addictive Behaviors. 2021; 116: 106814. Doi: 10.1016/j.addbeh.2020.106814.
