# Supplementary material for: Predictors of perceived success in quitting smoking by vaping: A machine learning approach
Source: PLoS One. 2022 Jan 14;17(1):e0262407. doi: 10.1371/journal.pone.0262407 (PMC8759658; doi:10.1371/journal.pone.0262407)

**S2 Appendix.** Inspection and imputation of missing values

Using the R package “naniar”, we found that 22.9% of participants had some degree of missing data which amounted to 0.8% of total data being absent from the dataset. Furthermore, out of the 51 person-level characteristics, 22 or 43.1% of these variables had missing responses, where two variables, including the preferred nicotine strength used in vaping (nic) and level of motivation to quit smoking (mot) contained missing values that exceeded 5%. The remaining variables had missingness<2%. In order to apply the multiple imputation by chained equation (mice) algorithm, we first verified the assumption of “missing at random” by visually inspecting the distribution of missing values in the figure on the next page. This assumption was deemed to be plausible.

Since all 51 person-level characteristics were categorical, we applied the Linear Discriminant Analysis (LDA) method to generate predicted values for imputation. For each variable, we used all the remaining variables as predictors except for four variables, including the two variables with missing values>5% (nicotine strength and motivation to quit); the VES; and the outcome variable (status of perceived success in smoking cessation by the use of e-cigarettes). Five imputed copies were independently generated. We used the first copy in primary analysis and the remaining four in sensitivity analysis.

Visual inspection of missing values in the dataset (column=variable, row=observation)


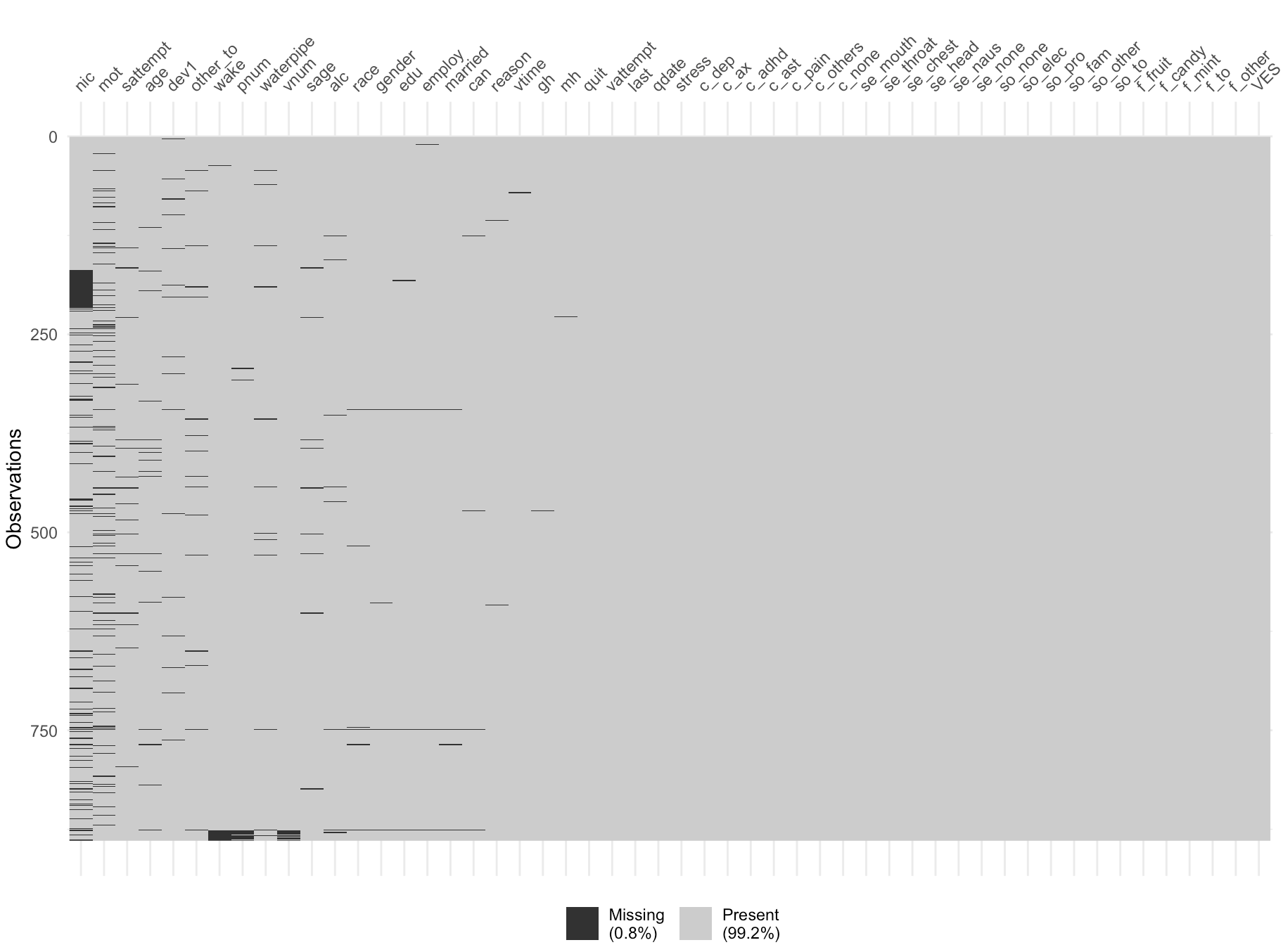

Supplement: S2 Appendix — (DOCX) [file pone.0262407.s004.docx]
