# Supplementary material for: Predictors of perceived success in quitting smoking by vaping: A machine learning approach
Source: PLoS One. 2022 Jan 14;17(1):e0262407. doi: 10.1371/journal.pone.0262407 (PMC8759658; doi:10.1371/journal.pone.0262407)
Supplement: S3 Appendix — (DOCX) [file pone.0262407.s005.docx]

**S3 Appendix.** Variable selection

To identify a subset of the most relevant predictors of perceived success in quitting smoking by vaping from the full set of 51 candidate predictors, we applied the following rules so that a predictor is selected into model training if:

(1) it is one of the six sociodemographic variables (age, gender, education, employment, marital status and race) OR

(2) it is selected by a Lasso logistic regression model.

In a Lasso logistic model, coefficient estimates $\hat{\beta_{0}}$,…, $\hat{\beta_{k}}$, were determined to minimize a cost function that comprised the negative log-likelihood function plus a term representing the total magnitudes of coefficient estimates multiplied by a unknown tuning parameter, $\lambda$:

$$L=\sum_{i=1}^{n} [-y_{i}(\beta_{0}+\beta_{1}x_{i1}+\ldots+\beta_{k}x_{\mathrm{ik}})+\log\left( 1+\exp\left( \beta_{0}+\beta_{1}x_{i1}+\ldots+\beta_{k}x_{\mathrm{ik}} \right)) \right]+ \lambda\sum_{j=1}^{k} |\beta_{j}|$$

The value of $\lambda$ ($\lambda>0$) determines the severity of penalty placed onto the number of variables with a non-zero coefficient and the magnitude of their coefficients. By choosing an optimal value of $\lambda$, the set of coefficient estimates reaches the best trade-off between adequate model fit and a manageable set of core predictors (i.e., a sparse estimation where only a small group of predictors have a non-zero coefficient). In order to locate this optimal $\lambda$ value, a ten-fold cross-validation procedure was performed on the full dataset (n=889). To do so, we randomly partitioned the data into ten equal-sized parts and set up a wide range of candidate $\lambda$ values to be searched (between 10^-5^ and 10^5^). At each iteration, the first nine parts of the partitioned data were used to estimate $\hat{\beta_{0}}$,…, $\hat{\beta_{k}}$ by minimizing the cost function presented above using each of the candidate $\lambda$ value. Then, using data from the remaining part, the $\lambda$ value that was associated with the set of coefficient estimates $\hat{\beta_{0}}$,…, $\hat{\beta_{k}}$ that minimized the negative log-likelihood function (or equivalently, maximized the log-likelihood function) was identified. This procedure was performed 10 times and the average $\lambda$ value pooled from the 10 iterations was considered to be optimal and used in the variable selection.

Using the R package “glmnet”, the cross-validated negative log-likelihood was minimized when $\lambda=0.006064329$. Using this $\lambda$ value, the table on the next page shows the coefficient estimate of each variable (where “.” Indicated a zero-coefficient).

| **Variables** | **Estimated coefficient in Lasso logistic model using** $\boldsymbol{\lambda=0.006064329}$ |
| --- | --- |
| (Intercept) | -3.0505807 |
| sage1 | -0.0435801 |
| gender1 | -0.2275739 |
| edu1 | . |
| edu2 | -0.0759776 |
| employ1 | 0.2956832 |
| married1 | 0.03811459 |
| married2 | . |
| race1 | -0.4194376 |
| vtime1 | 0.50686765 |
| reason1 | 0.92155106 |
| vattempt1 | -0.6458916 |
| vattempt2 | -0.8167827 |
| vattempt3 | -1.8065841 |
| last1 | -0.9050613 |
| last2 | 0.69390361 |
| mot1 | 0.47923454 |
| qdate1 | -0.2421614 |
| gh1 | -0.3566195 |
| gh2 | . |
| mh1 | . |
| mh2 | . |
| stress1 | . |
| stress2 | -0.092797 |
| c_dep1 | 0.01365722 |
| c_ax1 | -0.024932 |
| c_adhd1 | 0.13306494 |
| c_ast1 | -0.1769492 |
| c_pain1 | -0.2772131 |
| c_others1 | 0.14646741 |
| c_none1 | 0.1921221 |
| se_mouth1 | 0.32353984 |
| se_throat1 | . |
| se_chest1 | 0.11940484 |
| se_head1 | . |
| se_naus1 | 0.1808293 |
| se_none1 | -0.1384229 |
| so_none1 | 0.42869245 |
| so_elec1 | . |
| so_pro1 | . |
| so_fam1 | 0.08447508 |
| so_other1 | . |
| so_to1 | 0.38569815 |
| vnum1 | 0.23849836 |
| pnum1 | -0.0015672 |
| pnum2 | -0.0303843 |
| wake1 | 0.1891051 |
| wake2 | -0.3960882 |
| dev11 | 0.35824323 |
| f_fruit1 | . |
| f_candy1 | . |
| f_mint1 | . |
| f_to1 | . |
| f_other1 | 0.01952878 |
| nic1 | -0.5865511 |
| nic2 | -0.4093289 |
| can1 | -0.0118469 |
| can2 | 0.31288882 |
| alc1 | -0.0062515 |
| waterpipe1 | -0.5847374 |
| other_to1 | -0.0052643 |
| VES.1 | 1.83557325 |
| VES.2 | -0.0033833 |
| VES.3 | . |
| sattempt1 | 0.51126547 |
| age1 | . |
| age2 | . |
| age3 | -0.2758795 |

Hence, the following dummy variables were determined to be unimportant by the Lasso logistic regression procedure: edu1, married2, gh2, mh1, mh2, stress1, se_throat1, se_head1, so_elec1, so_prob1, so_other1, f_fruit1, f_candy1, f_mint1, f_to1, VES.3, age1 and age2. We did not exclude a dummy variable if it was associated with a feature with >2 levels and there were other dummy variables of this feature with a non-zero coefficient. Hence, the Lasso logistic regression procedure excluded 9 features (predictors): se_throat1, se_head1, so_elec1, so_prob1, so_other1, f_fruit1, f_candy1, f_mint1 and f_to1.
