# Supplementary material for: Predictors of perceived success in quitting smoking by vaping: A machine learning approach
Source: PLoS One. 2022 Jan 14;17(1):e0262407. doi: 10.1371/journal.pone.0262407 (PMC8759658; doi:10.1371/journal.pone.0262407)
Supplement: S4 Appendix — (DOCX) [file pone.0262407.s006.docx]

**S4 Appendix.** Train and tune a gradient boosting machine

**Internal SMOTE resampling**

In our data, the number of self-reported successful vaping-assisted quitters is significantly greater than that of unsuccessful quitters, with a ratio of roughly 1:4. This imbalance of outcomes raises concerns on weak model performance since classification algorithms are developed on the basis of reducing the mis-classification rate. Hence, the model is more likely to declare a majority case (in our case, an unsuccessful quitter) than a minority case (a successful quitter), which leads to poor sensitivity in detecting true positives. To mitigate this risk, we applied the SMOTE method to resample data on the training set using the “SMOTE” function in the R package “DMwR”. This method relies on the nearest neighbor technique to generate synthetic samples of the minority cases and then randomly under-sample the majority cases to achieve a balanced sample. In our training set (n=623), there are 501 unsuccessful quitters and 122 successful quitters. For each successful quitter, we created another 3 synthetic successful quitters to the training set using the nearest neighbor method (k=5), increasing the overall size of this class to 122*4=488 (366 new synthetic cases). We then randomly selected 500 out of the 501 unsuccessful quitters into the final balanced training set, achieving a size ratio of roughly 1:1.

**Gradient boosting machine (GBM)**

The GBM is an ensemble machine learning algorithm where many classification tree models are used jointly to produce prediction. Motivation of an ensemble model is that weak learners, i.e., simple tree models with poor performance, could be converted into a single strong model that is able to achieve high predictive accuracy and robust performance. To train a GBM, new tree models are added one at a time while existing trees are left unchanged. Individual tree split is determined by the Gini Index. A gradient descent procedure is used to minimize the objective function (i.e., a logarithm loss function) when adding trees.

**Model training**

In the default training, we assumed 5000 tree models were included in the GBM, each had an interaction depth of 6 with a minimum of 5 samples in a leaf node. A learning rate of 0.01 was assumed, meaning that the contribution of each additional tree was added to the overall prediction by a multiplicative factor of 0.01. For example, suppose the first tree predicts a smoker has 70% of chance to be a quitter and the second tree predicts this probability to be 99%. Then the prediction of the GBM is updated to be: 0.70 + 0.01 * 0.99 = 0.71.

Using these default settings, the 5000 tree models were sequentially developed using a gradient descent method to minimize the log loss function using data from the training set. This training procedure was conducted using a 3-fold cross-validation method: first, the training data was randomly split into three equal-size parts. Using the first part, we developed a GBM that minimized the log loss function and predicted the value of the log loss function using data from the remaining two parts. We repeated this procedure for each of the three parts and estimated the average log loss to be the overall performance of this GBM on the training set.

**Performance of the model on the training set**

The default GBM model fitted well on the training set (AUC=0.9996), but its performance was significantly poorer on the testing set (AUC=0.806), which was likely a result of overfitting due to excessive number of tree models (n=5000).

**Parameter tuning on the training set**

To mitigate model overfitting, we performed a series of “tuning” procedures to identify the optimal value for the four parameters of GBM that we had previously set at default: number of trees, interaction depth of trees (the maximum nodes or splits of each tree model), minimum number of observations in a leaf node and learning rate. First, we assumed the number of trees was 5000 and applied a grid search method to locate optimal values for the other three parameters. This procedure yielded an optimal GBM with 5000 trees with depth of 4 and a minimum of 5 observations in the leaf node, and a learning rate of 0.01.

To reduce the number of trees, we searched for a smaller tree number that enabled “early stopping” of model iteration. This number was identified to be when the classification error on the testing data started to increase while the performance of model continued to improve on the training data (which indicated overfitting on the training set that did not result in true improvement of model performance outside of the training data). Two methods were applied, including a 10-fold cross-validation and out-of-bag (OOB) sampling. The two methods gave 279 and 150 trees, respectively (see a plot on the next page). Since the OOB method often underestimates the number of trees, we preceded the analysis with 279 trees. Hence, our final model consisted of 279 trees each with a depth of 4 and a minimum of 5 observations in the leaf node and had a learning rate of 0.01.

We also considered a stochastic GBM where only a proportion of training set (between 65%-95%) was used for developing individual tree models. However, this model did not achieve better performance with the simple GBM, and thereby was not considered in further analysis.
