# Supplementary material for: Predictors of perceived success in quitting smoking by vaping: A machine learning approach
Source: PLoS One. 2022 Jan 14;17(1):e0262407. doi: 10.1371/journal.pone.0262407 (PMC8759658; doi:10.1371/journal.pone.0262407)

**S5 Appendix.** Procedures and results of sensitivity analysis

**Procedure 1: Assess the model on the remaining 4 imputed data copies**

We repeated model training, tuning, and testing using the other 4 imputed data copies (copies 2-5) and reported their model performance below:

| **Imputed data copy** | **Testing**  **AUC** | **Testing sensitivity** | **Testing**  **specificity** | **Testing accuracy** |
| --- | --- | --- | --- | --- |
| **Copy 1 (primary)** | 0.865 | 0.851  (0.798-0.902) | 0.750  (0.614-0.863) | 0.831  (0.780-0.874) |
| **Copy 2** | 0.864 | 0.851  (0.801-0.899) | 0.731  (0.592-0.838) | 0.827  (0.776-0.870) |
| **Copy 3** | 0.865 | 0.855  (0.804-0.903) | 0.789  (0.654-0.897) | 0.842  (0.793-0.884) |
| **Copy 4** | 0.869 | 0.808  (0.754-0.859) | 0.769  (0.630-0.871) | 0.801  (0.748-0.847) |
| **Copy 5** | 0.855 | 0.841  (0.790-0.889) | 0.731  (0.594-0.843) | 0.820  (0.768-0.864) |

Hence, the performance of GBM did not differ by the use of different data copies. We additionally used these models to identify the top 5 most important predictors and summarized the results below:

| **Imputed data copy** | **First predictor** | **Second predictor** | **Third predictor** | **Forth predictor** | **Fifth predictor** |
| --- | --- | --- | --- | --- | --- |
| **Copy 1 (primary)** | VES  (100%) | Vattempt  (39.0%) | Age  (21.9%) | Vtime  (16.8%) | Wake  (15.8%) |
| **Copy 2** | VES  (100%) | Vattempt  (43.9%) | Age  (19.1%) | Vtime  (18.0%) | Wake  (17.5%) |
| **Copy 3** | VES  (100%) | Vattempt  (37.9%) | Age  (21.3%) | Wake  (15.9%) | Vtime  (14.4%) |
| **Copy 4** | VES  (100%) | Vattempt  (44.9%) | Age  (26.4%) | Vtime  (22.8%) | Wake  (19.4%) |
| **Copy 5** | VES  (100%) | Vattempt  (47.2%) | Age  (18.1%) | Vtime  (16.7%) | Wake  (15.6%) |

The only difference was the order of “vtime” and “wake” in one of the five iteration where the importance score of “wake” exceeded that of “vtime”.

**Procedure 2: Assess a parsimonious model trained using only the top five predictors**

We repeated model training, tuning, and testing using only the five most important predictors: the VES, quit attempts by vaping, age, having vaped 100 times and the time after waking up to vape. Performance of this parsimonious model on the testing set is summarized below:

| **Models** | **Testing**  **AUC** | **Testing sensitivity** | **Testing**  **specificity** | **Testing accuracy** |
| --- | --- | --- | --- | --- |
| **Full model** | 0.865 | 0.851  (0.798-0.902) | 0.750  (0.614-0.863) | 0.831  (0.780-0.874) |
| **Parsimonious model** | 0.825 | 0.664  (0.609-0.735) | 0.827  (0.704-0.924) | 0.700  (0.636-0.750) |


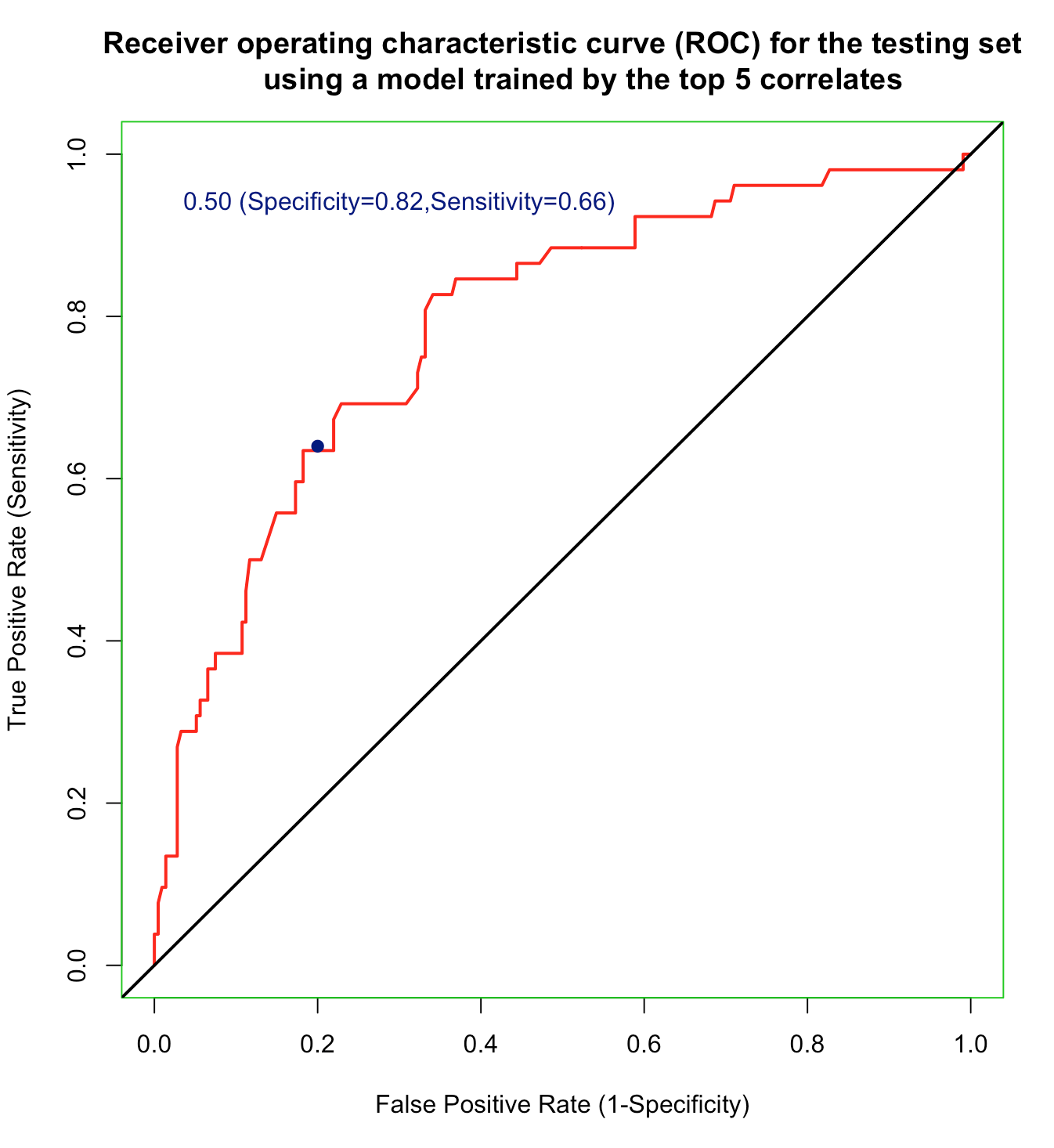


We then estimated the importance of the five predictors using this parsimonious model and the results were large consistent with findings from the full model:

| **Models** | **First predictor** | **Second predictor** | **Third predictor** | **Forth predictor** | **Fifth predictor** |
| --- | --- | --- | --- | --- | --- |
| **Full model** | VES  (100%) | Vattempt  (39.0%) | Age  (21.9%) | Vtime  (16.8%) | Wake  (15.8%) |
| **Parsimonious model** | VES  (100%) | Vattempt  (58.6%) | Age  (19.8%) | Vtime  (17.8%) | Wake  (17.7%) |


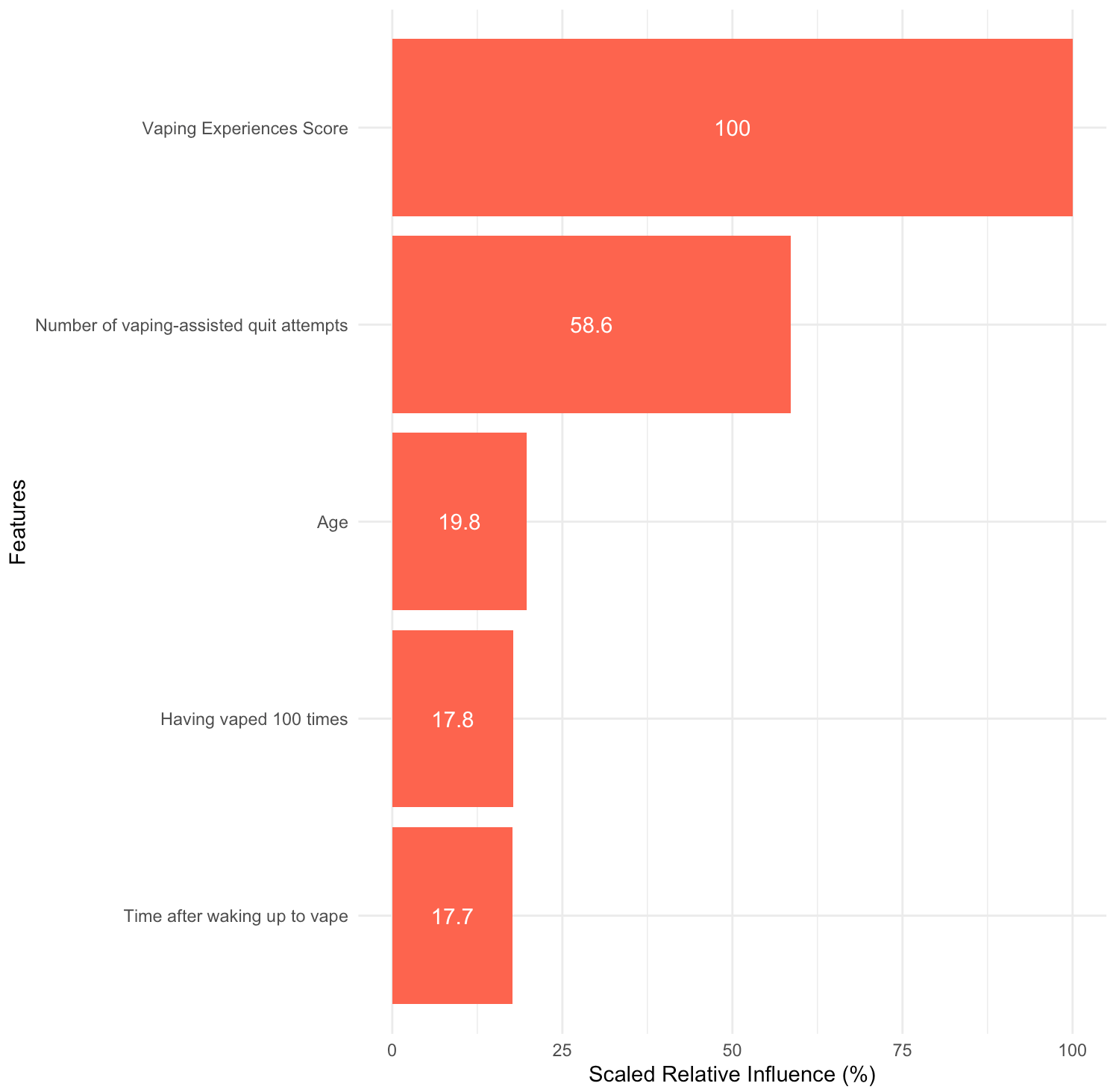


**Procedure 3: Assessing a model excluding the VES from training**

Excluding the VES from the training procedure, another GBM model was trained and tuned using the same resample training set. We compared its performance on the testing set with the full model and presented its ROC below:

| **Models** | **Testing**  **AUC** | **Testing sensitivity** | **Testing**  **specificity** | **Testing accuracy** |
| --- | --- | --- | --- | --- |
| **Full model** | 0.865 | 0.851  (0.798-0.902) | 0.750  (0.614-0.863) | 0.831  (0.780-0.874) |
| **Model excluding the VES** | 0.772 | 0.463  (0.399-0.532) | 0.846  (0.724-0.931) | 0.538  (0.476-0.599) |


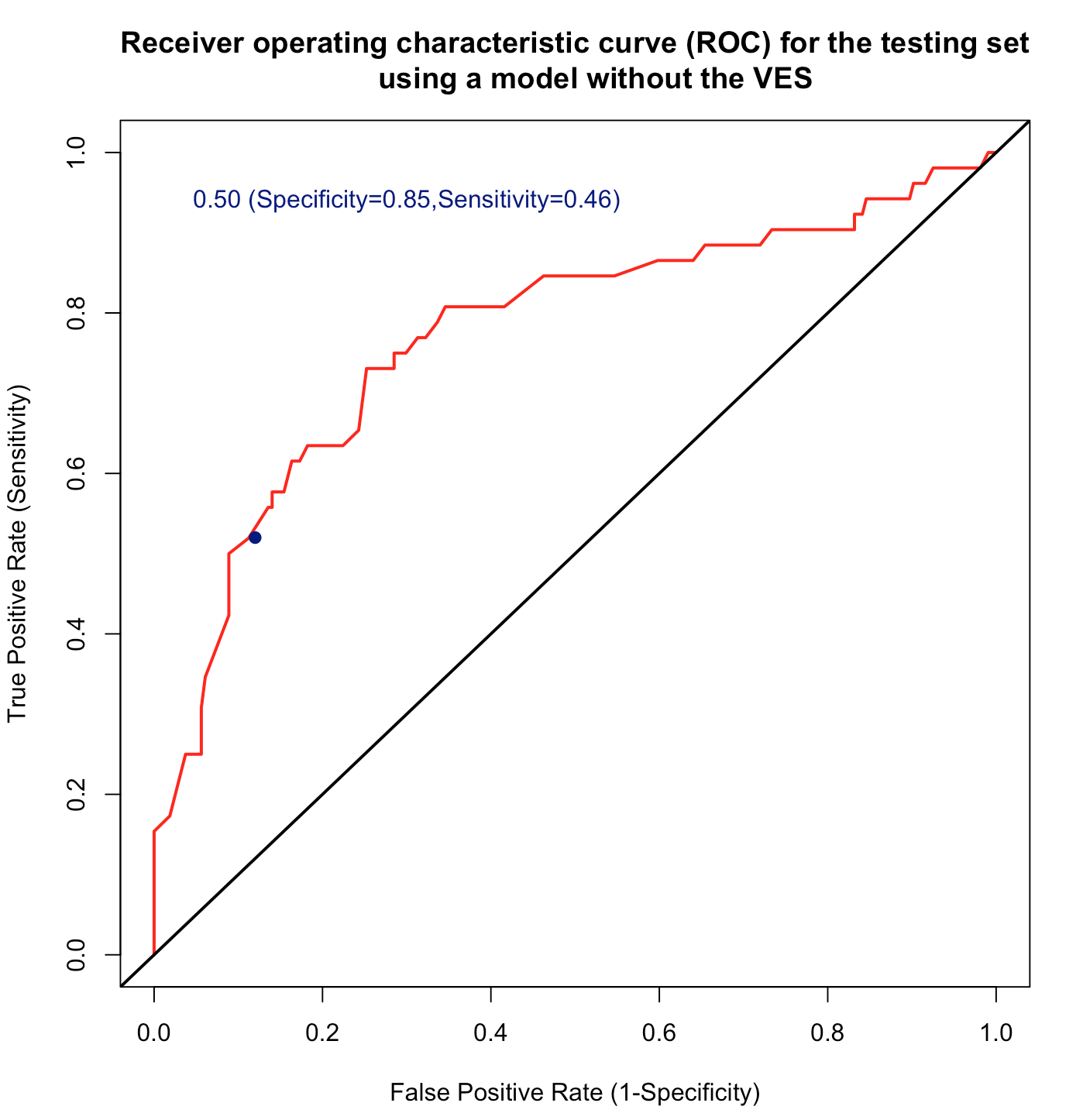


We then estimated the importance of the five predictors using this parsimonious model and the results were large consistent with findings from the full model:

| **Models** | **First predictor** | **Second predictor** | **Third predictor** | **Forth predictor** | **Fifth predictor** |
| --- | --- | --- | --- | --- | --- |
| **Full model** | VES  (100%) | Vattempt  (39.0%) | Age  (21.9%) | Vtime  (16.8%) | Wake  (15.8%) |
| **Model excluding the VES** | Vattempt  (100%) | Age  (55.7%) | Vtime  (51.7%) | Dev  (45.6%) | Wake  (37.4%) |


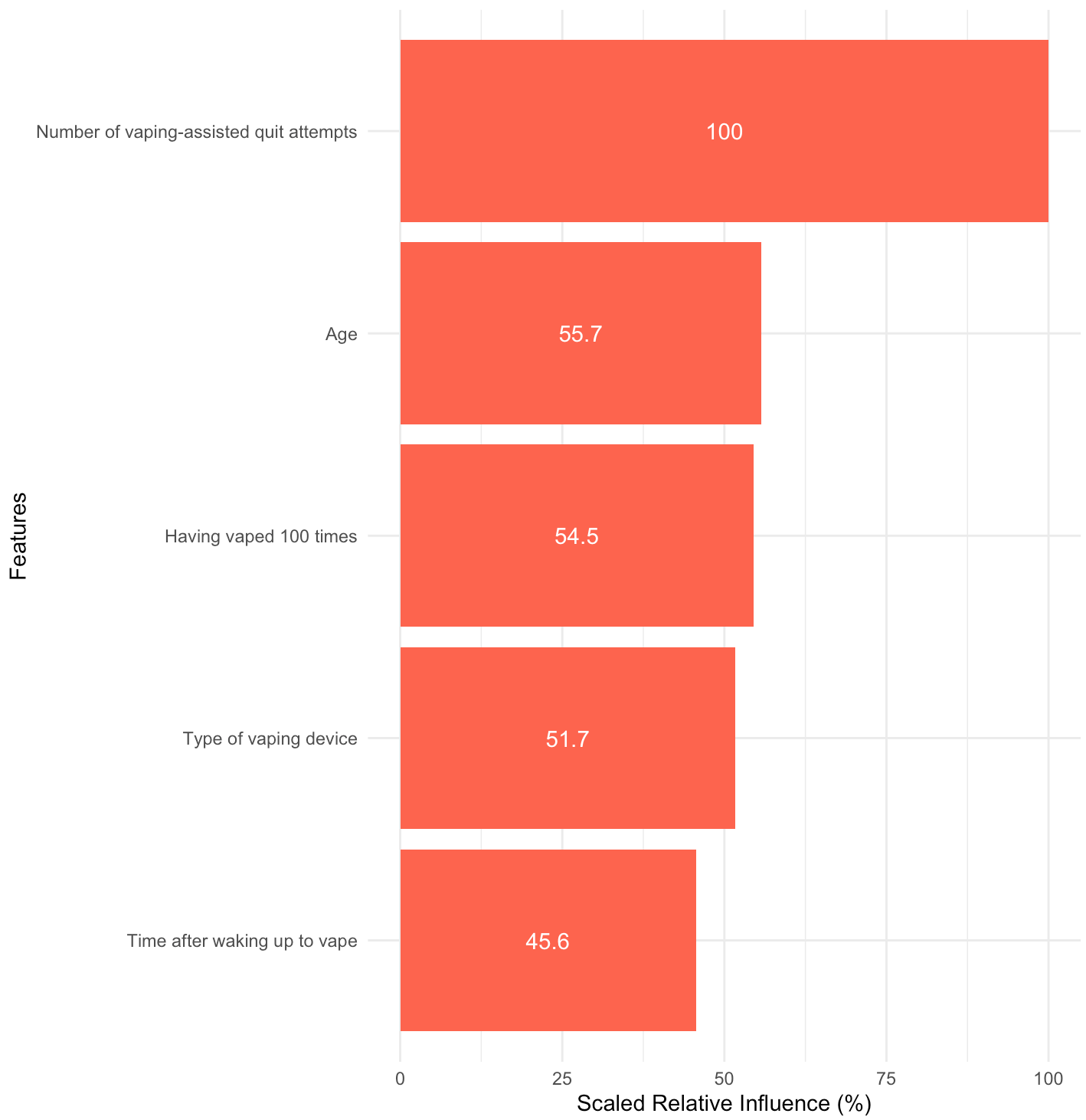


**Procedure 4: Multivariable logistic regression on data from the training set**

| **Variables** | **Reference level** | **OR** | **95% CI** | **P-value** |
| --- | --- | --- | --- | --- |
| **Participant age**  30-39  40-49  50+ | Age between 20-29 | 0.860  0.407  0.212 | 0.370-1.994  0.110-1.406  0.059-0.722 | 0.725  0.165  0.015** |
| **Started smoking at or after age 18** | Started smoking by 18 | 0.634 | 0.301-1.402 | 0.276 |
| **Non-female gender** | Identified as a female | 0.634 | 0.285-1.392 | 0.258 |
| **Highest education**  College diploma  University or above | High school or below | 0.501  0.284 | 0.213-1.155  0.094-0.813 | 0.107  0.021* |
| **Employed/self-employed** | Unemployed | 1.622 | 0.620-4.422 | 0.332 |
| **Marital status**  Married/living with a partner  Divorced/separated/widowed | Single/never married | 1.417  1.222 | 0.673-3.008  0.319-4.395 | 0.359  0.762 |
| **Non-white race** | Being white | 0.829 | 0.313-2.108 | 0.698 |
| **Having vaped 100 times** | Having vaped less than 100 times | 2.653 | 1.119-6.553 | 0.029* |
| **Started vaping to quit smoking** | Started vaping for other purposes | 6.232 | 2.068-21.778 | 0.002** |
| **Number of vaping-assisted quit attempts**  Second time  Third time  Fourth time or more | First time using vaping to quit smoking | 0.218  0.079  0.033 | 0.085-0.532  0.019-0.275  0.007-0.128 | 0.001**  0.0002***  <0.0001*** |
| **Time since the start of the last attempt to quit smoking by vaping**  Less than a month ago  More than 12 months ago | 1-12 months | 0.275  1.362 | 0.067-0.935  0.499-3.615 | 0.053  0.539 |
| **Highly motivated to quit smoking** | Not very or somewhat motivated | 2.124 | 0.954-4.964 | 0.072 |
| **Having a quit date** | Did not have a quit date | 0.569 | 0.260-1.220 | 0.150 |
| **Self-reported physical health**  Excellent or very good  Fair or poor | Good physical health | 0.215  0.528 | 0.065-0.643  0.219-1.251 | 0.008**  0.149 |
| **Self-reported mental health**  Excellent or very good  Fair or poor | Good mental health | 1.654  1.685 | 0.595-4.705  0.671-4.308 | 0.338  0.269 |
| **Self-reported stress level**  Quite stressful  Extremely stressful | Not very stressful | 0.807  0.385 | 0.346-1.869  0.130-1.101 | 0.617  0.078 |
| **Health conditions diagnosed before the last quit attempt**  Depression  Anxiety  ADHD  Asthma  Chronic pain  Other health condition | Did not have these health conditions | 1.362  0.732  2.251  0.241  0.586  3.489 | 0.505-3.721  0.257-2.092  0.774-6.598  0.059-0.860  0.133-2.244  1.301-9.773 | 0.542  0.558  0.136  0.036*  0.454  0.014** |
| **Did not have any health conditions** | Had some health conditions | 1.108 | 0.352-3.507 | 0.860 |
| **Side effects from vaping**  Mouth irritation  Throat irritation  Chest irritation  Headache  Nausea/vomiting/lightheaded | Did not have these side effects | 1.773  0.373  1.585  1.622  1.068 | 0.636-4.941  0.116-1.198  0.593-4.166  0.620-4.298  0.439-2.611 | 0.271  0.095  0.351  0.324  0.885 |
| **Did not have any side effects from vaping** | Had some side effects | 0.207 | 0.050-0.821 | 0.026* |
| **Did not seek help when vaping to quit smoking** | Having used some help | 4.785 | 1.368-18.284 | 0.017** |
| **Methods used when vaping to quit smoking**  Electronic sources  Professional help  Family and friend support  Alternative therapies  Other tobacco products | Did not use these methods during a vaping-assisted cigarette quit attempt | 0.859  3.614  1.289  1.906  1.007 | 0.259-2.764  1.123-12.583  0.491-3.389  0.526-6.622  0.394-2.700 | 0.800  0.036*  0.605  0.314  0.989 |
| **Vape 10+ times daily** | Vape less than 10 times | 2.307 | 1.012-5.436 | 0.050 |
| **Typical puffs per vape**  5-9 puffs  10+ puffs | Less than 5 puffs | 1.023  1.051 | 0.462-2.247  0.416-2.642 | 0.956  0.916 |
| **Time after waking up to vape**  Within 15-60 minutes  Beyond an hour | Within 15 minutes after waking up | 1.397  0.415 | 0.619-3.169  0.145-1.129 | 0.420  0.091 |
| **Using a pod system** | Did not use a pod system | 1.987 | 0.865-4.640 | 0.420 |
| **Flavors used in vape**  Fruity  Candy  Mint  Tobacco  Other flavors | Not using these flavors | 0.391  0.894  1.307  0.531  0.528 | 0.155-0.960  0.377-2.106  0.555-3.072  0.207-1.313  0.138-1.829 | 0.042*  0.800  0.538  0.177  0.329 |
| **Typical nicotine strength**  0.5%-2.0%  2.1% or more | Not using any nicotine in vape or using at most 0.5% | 0.243  0.272 | 0.098-0.576  0.088-0.793 | 0.002***  0.019** |
| **Type of cannabis user**  Daily/almost daily user  Occasional user | Have never used cannabis | 0.522  2.491 | 0.205-1.289  1.074-5.923 | 0.163  0.035* |
| **Daily or occasional alcohol drinker** | Not a drinker | 1.044 | 0.432-2.559 | 0.924 |
| **Using waterpipes** | Did not use waterpipes | 0.844 | 0.188-3.286 | 0.814 |
| **Using other tobacco products** | Did not use other tobacco products | 0.176 | 0.026-0.947 | 0.057 |
| **Vaping Experiences Score**  Fair  Good  Excellent | Poor experiences | 2.818  7.593  15.660 | 1.616-5.075  2.475-33.171  5.671-52.801 | 0.0004***  0.002***  <0.0001*** |
| **Having tried quitting smoking for at least 6 times by any means in life** | Having tried at most 5 times | 1.951 | 0.856-4.519 | 0.114 |

Notes: We used *, ** and *** to denote statistical significance levels of 5%, 1% and 0.1%.

OR, odds ratio; CI, confidence interval. ADHD, attention deficit hyperactivity disorder.

Using this logistic model, we predicted the status of vaping-assisted smoking cessation on data from the testing set. Performance of this model was compared with the full GBM model below:

| **Models** | **Testing**  **AUC** | **Testing sensitivity** | **Testing**  **specificity** | **Testing accuracy** |
| --- | --- | --- | --- | --- |
| **Full GBM model** | 0.865 | 0.851  (0.798-0.902) | 0.750  (0.614-0.863) | 0.831  (0.780-0.874) |
| **Logistic model estimated on the training data** | 0.701 | 0.940  (0.910-0.979) | 0.248  (0.147-0.362) | 0.808  (0.764-0.848) |


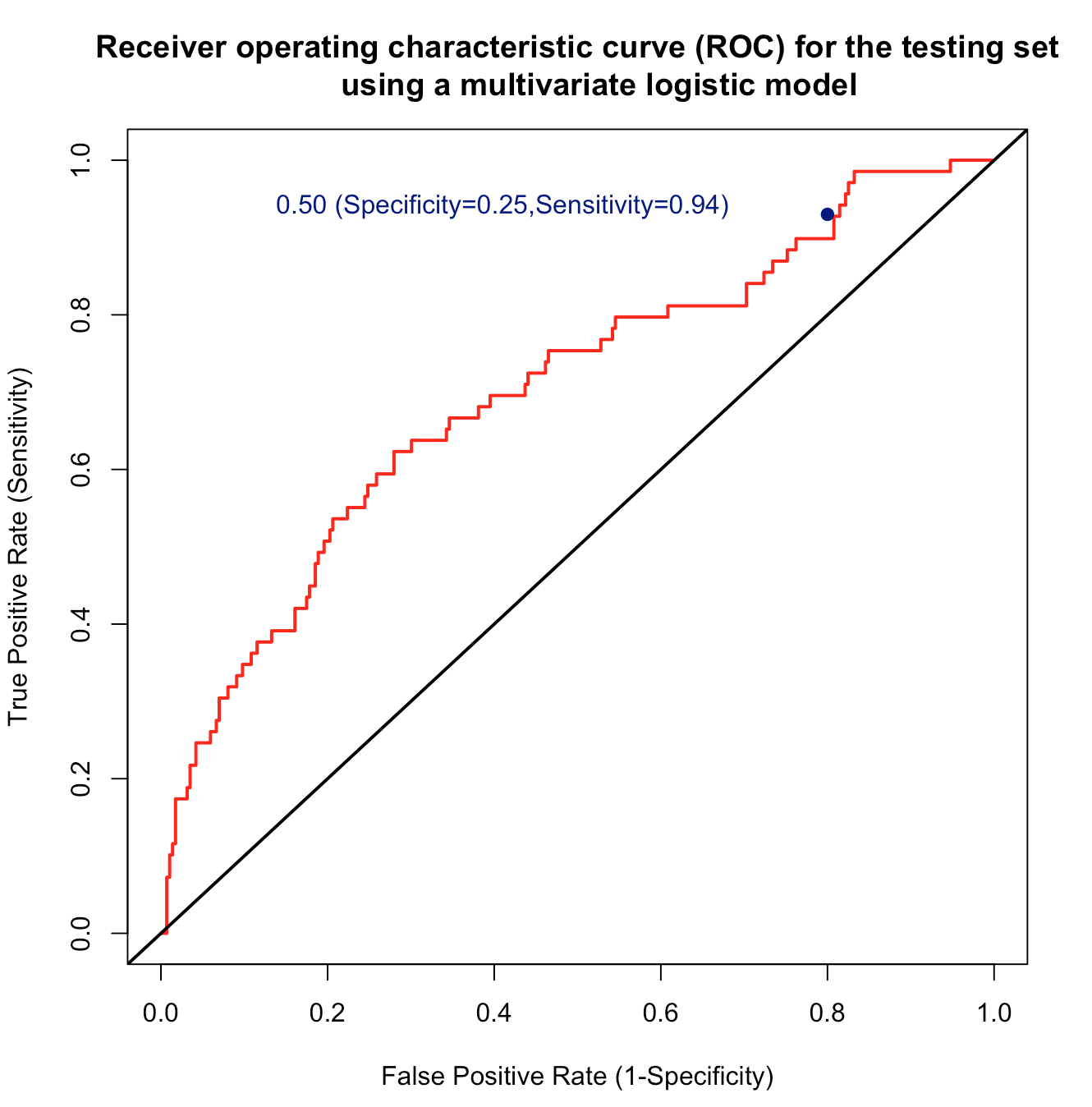

Supplement: S5 Appendix — (DOCX) [file pone.0262407.s007.docx]
