# Supplementary material for: Predictors of perceived success in quitting smoking by vaping: A machine learning approach
Source: PLoS One. 2022 Jan 14;17(1):e0262407. doi: 10.1371/journal.pone.0262407 (PMC8759658; doi:10.1371/journal.pone.0262407)
Supplement: S1 File — (PDF) [file pone.0262407.s008.pdf]

RECIG-WP Vape to Quit Survey - Part 2

Codebook

Data Dictionary Codebook

09/12/2019 2:43pm

Collapse all instruments

#	Variable / Field Name	Field Label<br>*Field Note*	Field Attributes (Field Type, Validation, Choices, Calculations, etc.)
Instrument: **Vaping history** (vaping_history)			Collapse
1	record_id	Record ID	text
2	sp_study_id	Study ID	text<br>Custom alignment: LV<br>Field Annotation: @HIDDEN
3	ecig_intro	In this study, vaping devices are products that use e-liquid with or without nicotine. They are also known as electronic cigarettes, e-cigarettes, vapes, pod systems (e.g., JUUL, Vype), mods and hookah pens. Herbal vapourizers and heated tobacco products (e.g., IQOS, Ploom, glo) should NOT be included when answering these questions unless otherwise stated.	descriptive
4	vstat	How often do you currently vape?	radio, Required<br>1 Daily or almost daily<br>2 Less than daily, but at least once a week<br>3 Less than weekly, but at least once a month<br>4 Less than monthly<br>5 Not at all<br>Custom alignment: LV
5	vbh3<br>Show the field ONLY if:<br>[vstat] = '4' or [vstat] = '5'	When was the LAST time you vaped?	radio<br>1 Less than one month ago<br>2 1-6 months ago<br>3 7-12 months ago<br>4 More than 12 months ago<br>Custom alignment: LV
6	lt_vape	How many times in your life have you vaped?	radio<br>1 Less than 10<br>3 11 to 20<br>4 21 to 50<br>5 51 to 100<br>6 More than 100<br>Custom alignment: LV
7	vbh1	What was the MAIN reason you first started vaping?	radio<br>1 To quit smoking cigarettes<br>2 To reduce the number of cigarettes I smoke<br>3 Some other reason (specify)<br>Custom alignment: LV
8	vbh1_specify<br>Show the field ONLY if:<br>[vbh1]= '3'	Please specify the MAIN reason you first started vaping.	text<br>Custom alignment: LV

9	vbh2	How many times have you EVER tried vaping to quit smoking cigarettes?	radio<br>0 None<br>1 1<br>2 2<br>3 3<br>4 4<br>5 5 or more<br><br>Custom alignment: LV
10	vbh4	How long ago did you LAST start vaping to quit smoking cigarettes?	radio<br>1 Less than one month ago<br>2 1-6 months ago<br>3 7-12 months ago<br>4 More than 1 year ago<br><br>Custom alignment: LV
11	sb7	Over the PAST 12 months, would you say that vaping has helped you be...?	radio<br>1 Not at all successful at cutting down smoking cigarettes<br>2 Somewhat successful at cutting down smoking cigarettes<br>3 Very successful at cutting down smoking cigarettes<br>4 Completely helped me quit smoking cigarettes<br><br>Custom alignment: LV
12	vaping_history_complete	Section Header: *Form Status*<br>Complete?	dropdown<br>0 Incomplete<br>1 Unverified<br>2 Complete
Instrument: **Concept Mapping Items** (concept_mapping_items) Collapse			
13	cm_pa_intro	This section has a list of statements that have been reported by people vaping to quit smoking.<br><br>This first list reflects some features about vaping. On a scale of 1 to 7, where 1='Not at all true for me' and 7='Extremely true for me', rate how true or not true each statement relates to your experience while vaping to quit.	descriptive
14	cm_pa1	Vaping doesn't leave a horrible taste in my mouth	radio (Matrix)<br>1 1 Not at all true for me<br>2 2<br>3 3<br>4 4<br>5 5<br>6 6<br>7 7 Extremely true for me

15	cm_pa2	I like the number of nicotine strengths available	radio (Matrix)<br>1 | 1 Not at all true for me<br>2 | 2<br>3 | 3<br>4 | 4<br>5 | 5<br>6 | 6<br>7 | 7 Extremely true for me
16	cm_pa3	When I vape I can take as many or as few puffs as I want. With cigarettes I often smoked more than I wanted because I was trying to finish the whole cigarette	radio (Matrix)<br>1 | 1 Not at all true for me<br>2 | 2<br>3 | 3<br>4 | 4<br>5 | 5<br>6 | 6<br>7 | 7 Extremely true for me
17	cm_pa4	With vaping, it is easier to lower my amount of nicotine intake than it was to cut down on the how many cigarettes I used to smoke	radio (Matrix)<br>1 | 1 Not at all true for me<br>2 | 2<br>3 | 3<br>4 | 4<br>5 | 5<br>6 | 6<br>7 | 7 Extremely true for me
18	cm_pa5	Vaping is easier than real cigarettes	radio (Matrix)<br>1 | 1 Not at all true for me<br>2 | 2<br>3 | 3<br>4 | 4<br>5 | 5<br>6 | 6<br>7 | 7 Extremely true for me
19	cm_pa6	I really like the flavours	radio (Matrix)<br>1 | 1 Not at all true for me<br>2 | 2<br>3 | 3<br>4 | 4<br>5 | 5<br>6 | 6<br>7 | 7 Extremely true for me
20	cm_pa7	I can vape almost anywhere	radio (Matrix)<br>1 | 1 Not at all true for me<br>2 | 2<br>3 | 3<br>4 | 4<br>5 | 5<br>6 | 6<br>7 | 7 Extremely true for me

21	cm_pa8	Vaping costs less than smoking	radio (Matrix)<br>1 1 Not at all true for me<br>2 2<br>3 3<br>4 4<br>5 5<br>6 6<br>7 7 Extremely true for me
22	cm_pa9	I like my device because it is portable, durable, and comes in nice colours	radio (Matrix)<br>1 1 Not at all true for me<br>2 2<br>3 3<br>4 4<br>5 5<br>6 6<br>7 7 Extremely true for me
23	cm_pa10	Vaping satisfies the associated sensations related to smoking cigarettes. (e.g., the 'smoke', inhaling, the throat tickle, hand motions, and the oral fixation)	radio (Matrix)<br>1 1 Not at all true for me<br>2 2<br>3 3<br>4 4<br>5 5<br>6 6<br>7 7 Extremely true for me
24	cm_pa11	The vape store employees are very knowledgeable and helpful	radio (Matrix)<br>1 1 Not at all true for me<br>2 2<br>3 3<br>4 4<br>5 5<br>6 6<br>7 7 Extremely true for me<br>8 I have never been to a vape store
25	cm_sc_intro	The next list reflects some social experiences with vaping that have been reported by people vaping to quit. On a scale of 1 to 7, where 1='Not at all true for me' and 7='Extremely true for me', rate how true or not true each statement relates to your experience while vaping to quit.	descriptive
26	cm_sc1	People think that vapour is the same as secondhand cigarette smoke	radio (Matrix)<br>1 1 Not at all true for me<br>2 2<br>3 3<br>4 4<br>5 5<br>6 6<br>7 7 Extremely true for me

27	cm_sc2	People judge you for vaping	radio (Matrix)<br>1	1 Not at all true for me<br>2	2<br>3	3<br>4	4<br>5	5<br>6	6<br>7	7 Extremely true for me
28	cm_sc3	I feel stupid using my vape in public	radio (Matrix)<br>1	1 Not at all true for me<br>2	2<br>3	3<br>4	4<br>5	5<br>6	6<br>7	7 Extremely true for me
29	cm_sc4	People don't like me vaping around them	radio (Matrix)<br>1	1 Not at all true for me<br>2	2<br>3	3<br>4	4<br>5	5<br>6	6<br>7	7 Extremely true for me
30	cm_sc5	People often complain about the amount of vapour emitted by the devices and it makes it hard to use them in many places	radio (Matrix)<br>1	1 Not at all true for me<br>2	2<br>3	3<br>4	4<br>5	5<br>6	6<br>7	7 Extremely true for me
31	cm_po_intro	Section Header:<br>The next list reflects some outcomes people have reported while vaping to quit. On a scale of 1 to 7, where 1='Not at all true for me' and 7='Extremely true for me', rate how true or not true each statement relates to your experience while vaping to quit.	descriptive
32	cm_po1	I don't smell like cigarettes all the time	radio (Matrix)<br>1	1 Not at all true for me<br>2	2<br>3	3<br>4	4<br>5	5<br>6	6<br>7	7 Extremely true for me

33	cm_po2	I cut down on smoking cigarettes	radio (Matrix)<br>1 1 Not at all true for me<br>2 2<br>3 3<br>4 4<br>5 5<br>6 6<br>7 7 Extremely true for me
34	cm_po3	I feel less dirty when vaping	radio (Matrix)<br>1 1 Not at all true for me<br>2 2<br>3 3<br>4 4<br>5 5<br>6 6<br>7 7 Extremely true for me
35	cm_po4	By vaping, I don't have the guilt of smoking cigarettes	radio (Matrix)<br>1 1 Not at all true for me<br>2 2<br>3 3<br>4 4<br>5 5<br>6 6<br>7 7 Extremely true for me
36	cm_po5	I feel like I can breathe again	radio (Matrix)<br>1 1 Not at all true for me<br>2 2<br>3 3<br>4 4<br>5 5<br>6 6<br>7 7 Extremely true for me
37	cm_po6	I feel better	radio (Matrix)<br>1 1 Not at all true for me<br>2 2<br>3 3<br>4 4<br>5 5<br>6 6<br>7 7 Extremely true for me
38	cm_po7	I feel like it is worth taking care of my teeth again	radio (Matrix)<br>1 1 Not at all true for me<br>2 2<br>3 3<br>4 4<br>5 5<br>6 6<br>7 7 Extremely true for me

39	cm_po8	Vaping is better for my health than smoking	radio (Matrix)<br>1 1 Not at all true for me<br>2 2<br>3 3<br>4 4<br>5 5<br>6 6<br>7 7 Extremely true for me
40	cm_po9	I can smell things again	radio (Matrix)<br>1 1 Not at all true for me<br>2 2<br>3 3<br>4 4<br>5 5<br>6 6<br>7 7 Extremely true for me
41	cm_po10	I have more energy	radio (Matrix)<br>1 1 Not at all true for me<br>2 2<br>3 3<br>4 4<br>5 5<br>6 6<br>7 7 Extremely true for me
42	cm_po11	My sense of taste returned	radio (Matrix)<br>1 1 Not at all true for me<br>2 2<br>3 3<br>4 4<br>5 5<br>6 6<br>7 7 Extremely true for me
43	cm_rel_intro	Section Header:<br>The next list reflects statements about possible relationship experiences while vaping to quit. On a scale of 1 to 7, where 1='Not at all true for me' and 7='Extremely true for me', rate how true or not true each statement relates to your experience while vaping to quit.	descriptive
44	cm_rel1	My family members prefer me vaping because of the health benefits	radio (Matrix)<br>1 1 Not at all true for me<br>2 2<br>3 3<br>4 4<br>5 5<br>6 6<br>7 7 Extremely true for me

45	cm_rel2	I like that I can tell people I don't smoke	radio (Matrix)<br>1 1 Not at all true for me<br>2 2<br>3 3<br>4 4<br>5 5<br>6 6<br>7 7 Extremely true for me
46	cm_rel3	My family and friends are proud of me for quitting smoking through vaping	radio (Matrix)<br>1 1 Not at all true for me<br>2 2<br>3 3<br>4 4<br>5 5<br>6 6<br>7 7 Extremely true for me
47	cm_rel4	I prefer vaping because I don't like the stigma of smoking	radio (Matrix)<br>1 1 Not at all true for me<br>2 2<br>3 3<br>4 4<br>5 5<br>6 6<br>7 7 Extremely true for me
48	cm_rel5	I'm more accepted by my non-smoking friends when I vape	radio (Matrix)<br>1 1 Not at all true for me<br>2 2<br>3 3<br>4 4<br>5 5<br>6 6<br>7 7 Extremely true for me
49	cm_hlth_intro	The next list of statements reflects some other outcomes people have reported while vaping to quit. On a scale of 1 to 7, where 1='Not at all true for me' and 7='Extremely true for me', rate how true or not true each statement relates to your experience while vaping to quit.	descriptive
50	cm_hlth1	It's frustrating to have to vape outside and be around people smoking cigarettes - it makes me just want to smoke	radio (Matrix)<br>1 1 Not at all true for me<br>2 2<br>3 3<br>4 4<br>5 5<br>6 6<br>7 7 Extremely true for me

51	cm_hlth2	I worry about the effects of vaping on my health	radio (Matrix)<br>1 1 Not at all true for me<br>2 2<br>3 3<br>4 4<br>5 5<br>6 6<br>7 7 Extremely true for me
52	cm_hlth3	When I inhale too quickly, I burn my throat which turns into a coughing fit	radio (Matrix)<br>1 1 Not at all true for me<br>2 2<br>3 3<br>4 4<br>5 5<br>6 6<br>7 7 Extremely true for me
53	cm_hlth4	The vapour makes me choke at first and it is hard to breathe	radio (Matrix)<br>1 1 Not at all true for me<br>2 2<br>3 3<br>4 4<br>5 5<br>6 6<br>7 7 Extremely true for me
54	cm_hlth5	Vaping makes me dehydrated	radio (Matrix)<br>1 1 Not at all true for me<br>2 2<br>3 3<br>4 4<br>5 5<br>6 6<br>7 7 Extremely true for me
55	cm_hlth6	I find that vaping didn't work for me as it was too close to smoking	radio (Matrix)<br>1 1 Not at all true for me<br>2 2<br>3 3<br>4 4<br>5 5<br>6 6<br>7 7 Extremely true for me
56	cm_lim_intro	Section Header:<br>The final list of statements reflects some other experiences reported while vaping to quit. On a scale of 1 to 7, where 1='Not at all true for me' and 7='Extremely true for me', rate how true or not true each statement relates to your experience while vaping to quit.	descriptive

57	cm_lim1	I feel like I am replacing one habit with another	radio (Matrix)<br>1 1 Not at all true for me<br>2 2<br>3 3<br>4 4<br>5 5<br>6 6<br>7 7 Extremely true for me
58	cm_lim2	I feel like I had to constantly vape to feel satisfied	radio (Matrix)<br>1 1 Not at all true for me<br>2 2<br>3 3<br>4 4<br>5 5<br>6 6<br>7 7 Extremely true for me
59	cm_lim3	The fruit or candy flavours didn't satisfy my craving for a cigarette	radio (Matrix)<br>1 1 Not at all true for me<br>2 2<br>3 3<br>4 4<br>5 5<br>6 6<br>7 7 Extremely true for me
60	cm_lim4	Vaping didn't work to help me quit smoking	radio (Matrix)<br>1 1 Not at all true for me<br>2 2<br>3 3<br>4 4<br>5 5<br>6 6<br>7 7 Extremely true for me
61	cm_lim5	I'm unsure about where I can vape indoors	radio (Matrix)<br>1 1 Not at all true for me<br>2 2<br>3 3<br>4 4<br>5 5<br>6 6<br>7 7 Extremely true for me
62	cm_lim6	I'm apprehensive to enter brick and mortar vape shops / lounges	radio (Matrix)<br>1 1 Not at all true for me<br>2 2<br>3 3<br>4 4<br>5 5<br>6 6<br>7 7 Extremely true for me

63	cm_dev1	Vaping is expensive	radio (Matrix)<br>1 | 1 Not at all true for me<br>2 | 2<br>3 | 3<br>4 | 4<br>5 | 5<br>6 | 6<br>7 | 7 Extremely true for me
64	cm_dev2	The variety of devices available is confusing when you don't know what you're looking for	radio (Matrix)<br>1 | 1 Not at all true for me<br>2 | 2<br>3 | 3<br>4 | 4<br>5 | 5<br>6 | 6<br>7 | 7 Extremely true for me
65	cm_dev3	Cheap devices don't work as well	radio (Matrix)<br>1 | 1 Not at all true for me<br>2 | 2<br>3 | 3<br>4 | 4<br>5 | 5<br>6 | 6<br>7 | 7 Extremely true for me
66	cm_dev4	There's a learning curve to vaping and finding what juice/nicotine/device works best for me	radio (Matrix)<br>1 | 1 Not at all true for me<br>2 | 2<br>3 | 3<br>4 | 4<br>5 | 5<br>6 | 6<br>7 | 7 Extremely true for me
67	cm_dev5	I don't like having to replace the coil in my vape	radio (Matrix)<br>1 | 1 Not at all true for me<br>2 | 2<br>3 | 3<br>4 | 4<br>5 | 5<br>6 | 6<br>7 | 7 Extremely true for me
68	vq_experience	Please share any other experiences you've had while vaping to quit smoking.	notes<br>Custom alignment: LV
69	concept_mapping_items_complete	Section Header: *Form Status*<br>Complete?	dropdown<br>0 | Incomplete<br>1 | Unverified<br>2 | Complete
Instrument: **Behaviour Survey** (behaviour_survey)			Collapse
70	sb_intro	The next questions ask about cigarette smoking.	descriptive

71	smkstat	At the present time, do you smoke cigarettes?	radio, Required<br>1 Every day or almost every day<br>2 Occasionally<br>3 Not at all<br><br>Custom alignment: LV
72	lastcig<br><br>Show the field ONLY if:<br>[smkstat] = '2' or [smkstat] = '3'	When was the last time you smoked a cigarette, even a puff?	radio<br>1 Less than one month ago<br>2 1 to 2 months ago<br>3 3 to 4 months ago<br>4 5 to 6 months ago<br>5 7 to 12 months ago<br>6 More than 12 months ago<br><br>Custom alignment: LV
73	sb9<br><br>Show the field ONLY if:<br>([vstat]='1' or [vstat]='2') and ([smkstat]='1' or [smkstat] ='2')	Are you currently vaping to try and quit smoking cigarettes?	yesno<br>1 Yes<br>0 No<br><br>Custom alignment: LV
74	sb12<br><br>Show the field ONLY if:<br>[smkstat]='3'	Are you now smoke-free because you have completely quit smoking cigarettes by vaping?	yesno<br>1 Yes<br>0 No<br><br>Custom alignment: LV
75	sb1<br><br>Show the field ONLY if:<br>[smkstat]='1' or [lastcig]='1'	On how many of the last 30 days did you smoke cigarettes, even a puff? Enter a number between 0 and 30.	text (number, Min: 0, Max: 30)<br>Custom alignment: LV
76	sb2_s<br><br>Show the field ONLY if:<br>[smkstat]='1' or [smkstat]='2'	On the days that you smoke, approximately how many CIGARETTES do you smoke per day?	text (number, Min: 0, Max: 100)<br>Custom alignment: LV
77	sb3_s<br><br>Show the field ONLY if:<br>[smkstat]='1'	How soon after you wake up do you smoke your first cigarette of the day?	radio<br>1 0-5 minutes<br>2 6-15 minutes<br>3 16-30 minutes<br>4 31-60 minutes<br>5 61-120 minutes<br>6 More than 120 minutes<br><br>Custom alignment: LV
78	sb8	At what age did you start smoking regularly? Enter number only.	text (number, Min: 5, Max: 70)<br>Custom alignment: LV
79	lt_qa	How many times in your life have you made a serious attempt to quit smoking cigarettes? By serious, we mean making a conscious effort to stay off cigarettes for good. Enter number only.	text (number, Min: 0, Max: 30)<br>Custom alignment: LV
80	ecig_vq_intro<br><br>Show the field ONLY if:<br>[sb9]='1'	Section Header:<br>For these next questions, please think about your behaviours BEFORE you started your attempt to quit smoking by vaping.	descriptive
81	ecig_quit_intro<br><br>Show the field ONLY if:<br>[sb12]='1'	Congratulations on quitting smoking by vaping!<br><br>For these next questions, please think about your behaviours BEFORE you started your attempt to quit.	descriptive

82	ecig_intro2<br>Show the field ONLY if:<br>[sb9]<>'1' and [sb12]<>'1'	For these next questions, please think about your behaviours BEFORE you started your LAST attempt to quit smoking by vaping.	descriptive
83	sb4<br>Show the field ONLY if:<br>[smkstat]='2' or [smkstat]='3'	Before you started your attempt to quit, were you smoking cigarettes every day?	yesno<br>1 Yes<br>0 No<br>Custom alignment: LV
84	sb2_ns<br>Show the field ONLY if:<br>[sb4]='1'	Before starting your quit attempt, approximately how many CIGARETTES were you smoking each day?	text (number, Min: 0, Max: 100)<br>Custom alignment: LV
85	sb3_ns<br>Show the field ONLY if:<br>[sb4]='1'	Before starting your quit attempt, how soon after you woke up did you smoke your first cigarette of the day?	radio<br>1 0-5 minutes<br>2 6-15 minutes<br>3 16-30 minutes<br>4 31-60 minutes<br>5 61-120 minutes<br>6 More than 120 minutes<br>Custom alignment: LV
86	sb5	On a scale of 0 to 10, where 0="not at all motivated" and 10="extremely motivated", how motivated were you to quit smoking cigarettes?	slider<br>Slider labels: Not at all motivated<br>0,<br><br>5, Extremely motivated<br>10<br>Custom alignment: LH
87	vbh5	Before you started your quit attempt, did you set a quit date?	yesno<br>1 Yes<br>0 No<br>Custom alignment: LV
88	vbh6<br>Show the field ONLY if:<br>[vbh5] = '1'	In relation to your quit date, when did you start vaping?	radio<br>1 More than a month before I started my quit attempt<br>2 3-4 weeks before<br>3 1-2 weeks before<br>4 Less than 1 week before<br>5 The day I started my quit attempt<br>6 Sometime after I started my quit attempt<br>Custom alignment: LV
89	ghealth	How would you rate your overall HEALTH before your quit attempt started?	radio<br>1 Excellent<br>2 Very good<br>3 Good<br>4 Fair<br>5 Poor<br>Custom alignment: LV

90	mhealth	How would you rate your overall MENTAL HEALTH before your quit attempt started?	radio<br>1 Excellent<br>2 Very good<br>3 Good<br>4 Fair<br>5 Poor<br><br>Custom alignment: LV
91	pstress	How would you rate the overall level of STRESS in your life before your quit attempt started?	radio<br>1 Not at all stressful<br>2 Not very stressful<br>3 A bit stressful<br>4 Quite a bit stressful<br>5 Extremely stressful<br><br>Custom alignment: LV
92	condition	Before you started your attempt to quit smoking, were you being treated for, or had you been diagnosed with, any of the following? Check all that apply.	checkbox<br>1 condition___1 Depression<br>2 condition___2 Anxiety<br>3 condition___3 Attention Deficit Hyperactivity Disorder (ADHD)<br>4 condition___4 Alcohol problems<br>5 condition___5 Chronic lung disease (e.g., emphysema, chronic bronchitis)<br>6 condition___6 Asthma<br>7 condition___7 Chronic pain<br>8 condition___8 Diabetes<br>9 condition___9 Heart disease<br>10 condition___10 Lung cancer<br>11 condition___11 Other cancers<br>12 condition___12 Other condition (specify)<br>13 condition___13 None of the above<br><br>Custom alignment: LV<br>Field Annotation: @NONEOFTHEABOVE=13
93	condition_other<br>Show the field ONLY if: [condition(12)] = '1'	Please specify the other condition(s) you were being treated for, or had been diagnosed with, before your quit attempt started.	text<br>Custom alignment: LV
94	vq_intro<br>Show the field ONLY if: [sb9]<>"1" and [sb12]<>"1"	Section Header:<br>Thank you - you've completed a good part of the survey already!<br><br>The next questions ask about your specific experiences while vaping to quit in the PAST 12 MONTHS.	descriptive
95	vq_intro_2<br>Show the field ONLY if: [sb9]="1" or [sb12]="1"	Thank you - you've completed a good part of the survey already!<br><br>The next questions ask about your specific experiences while vaping to quit.	descriptive

<table>
<tr><td>96</td><td>vbh16</td><td>Have you EVER experienced any of the following negative side effects from vaping? Check all that apply.</td><td>checkbox
<table>
<tr><td>1</td><td>vbh16___1</td><td>Mouth irritation</td></tr>
<tr><td>2</td><td>vbh16___2</td><td>Throat irritation</td></tr>
<tr><td>3</td><td>vbh16___3</td><td>Chest irritation</td></tr>
<tr><td>4</td><td>vbh16___4</td><td>Cough</td></tr>
<tr><td>5</td><td>vbh16___5</td><td>Headache</td></tr>
<tr><td>6</td><td>vbh16___6</td><td>Nausea</td></tr>
<tr><td>7</td><td>vbh16___7</td><td>Lightheadedness</td></tr>
<tr><td>8</td><td>vbh16___8</td><td>Losing sense of taste</td></tr>
<tr><td>9</td><td>vbh16___9</td><td>Vomiting</td></tr>
<tr><td>10</td><td>vbh16___10</td><td>Other (specify)</td></tr>
<tr><td>11</td><td>vbh16___11</td><td>I haven't experienced any side effects</td></tr>
</table>
Custom alignment: LV
Field Annotation: @NONEOFTHEABOVE=11</td></tr>
<tr><td>97</td><td>vbh16_other
Show the field ONLY if:
[vbh16(10)] = '1'</td><td>Please specify any other negative side effects you have experienced.</td><td>text
Custom alignment: LV</td></tr>
<tr><td>98</td><td>vq1
Show the field ONLY if:
[sb9]<>"1" and [sb12]<>"1"</td><td>While vaping to quit, did you stop smoking cigarettes completely?</td><td>yesno
<table>
<tr><td>1</td><td>Yes</td></tr>
<tr><td>0</td><td>No</td></tr>
</table>
Custom alignment: LV</td></tr>
<tr><td>99</td><td>vq2
Show the field ONLY if:
[vq1] = '1'</td><td>How long were you able to stay completely smoke-free?</td><td>radio
<table>
<tr><td>1</td><td>Less than 1 day</td></tr>
<tr><td>2</td><td>More than a day but less than 1 week</td></tr>
<tr><td>3</td><td>1-2 weeks</td></tr>
<tr><td>4</td><td>3-4 weeks</td></tr>
<tr><td>5</td><td>1-2 months</td></tr>
<tr><td>6</td><td>3-4 months</td></tr>
<tr><td>7</td><td>5-6 months</td></tr>
<tr><td>8</td><td>7-12 months</td></tr>
<tr><td>9</td><td>More than 12 months</td></tr>
</table>
Custom alignment: LV</td></tr>
<tr><td>100</td><td>vq3
Show the field ONLY if:
[vq2] > '1' or [sb12]='1'</td><td>Once you started vaping, how long did you continue to smoke any cigarettes before you quit completely?</td><td>radio
<table>
<tr><td>1</td><td>Not at all</td></tr>
<tr><td>2</td><td>Less than 1 day</td></tr>
<tr><td>3</td><td>More than 1 day but less than 1 week</td></tr>
<tr><td>4</td><td>1-2 weeks</td></tr>
<tr><td>5</td><td>3-4 weeks</td></tr>
<tr><td>6</td><td>1-2 months</td></tr>
<tr><td>7</td><td>3-4 months</td></tr>
<tr><td>8</td><td>5-6 months</td></tr>
<tr><td>9</td><td>7 to 12 months</td></tr>
<tr><td>10</td><td>More than 12 months</td></tr>
</table>
Custom alignment: LV</td></tr>
</table>

101	vq4<br>Show the field ONLY if:<br>[vq1] = '0' or ([vq2]='1' or [vq2]='2') or (([smkstat]='1' or [smkstat]='2') and [sb9]='0')	While vaping to quit, did you reduce the number of cigarettes you smoked?	yesno<br>1 Yes<br>0 No<br>Custom alignment: LV
102	vq5<br>Show the field ONLY if:<br>[vq4] = '1'	Approximately how many cigarettes did you reduce per day?	text (number, Min: 0, Max: 75)<br>Custom alignment: LV
103	vq6<br>Show the field ONLY if:<br>[vq4] = '1'	How long did you vape before you started to reduce the number of cigarettes you smoked?	radio<br>1 Not at all<br>2 Less than 1 day<br>3 More than 1 day but less than 1 week<br>4 1-2 weeks<br>5 3-4 weeks<br>6 1-2 months<br>7 3-4 months<br>8 5-6 months<br>9 7 to 12 months<br>10 More than 12 months<br>Custom alignment: LV
104	vq7	What other resources or methods did you use when you LAST vaped to quit smoking? Check all that apply.	checkbox<br>1 vq7___1 I did not use any other resources or methods to help me quit<br>2 vq7___2 Heated tobacco (e.g., IQOS, Ploom, glo)<br>3 vq7___3 Telephone quitline (e.g., Smokers' Helpline)<br>4 vq7___4 Online quitting resources (e.g., Smokers' Helpline Online or other websites or blogs)<br>5 vq7___5 Text Messaging / Mobile Cessation Applications<br>6 vq7___6 Prescription medication(s) for smoking cessation such as Champix (varenicline), or Zyban/Wellbutrin (bupropion)<br>7 vq7___7 Nicotine replacement therapy (e.g., nicotine patch, gum, lozenge, inhaler)<br>8 vq7___8 Advice from health care professionals (e.g., doctor, pharmacist, nurse, dentist/hygienist)<br>9 vq7___9 Group or individual counselling<br>10 vq7___10 Support from friends and/or family<br>11 vq7___11 Public health unit/local cessation program<br>12 vq7___12 Other self-help resources<br>13 vq7___13 Alternative treatments (e.g., laser therapy, acupuncture, hypnosis, herbal remedies, etc.)<br>14 vq7___14 Other (specify)<br>Custom alignment: LV<br>Field Annotation: @NONEOFTHEABOVE=1

105	vq7_other<br><br>Show the field ONLY if:<br>[vq7(14)] = '1'	Please specify the other resources or methods you used the LAST time you vaped to try to quit.	text<br>Custom alignment: LV
106	sb6	When vaping to quit, what other forms of tobacco were you using? Check all that apply.	checkbox<br>1 sb6___1 Heated tobacco products (e.g., IQOS, Ploom, i-glo/glo)<br>2 sb6___2 Cigars, cigarillos, pipes<br>3 sb6___3 Smokeless tobacco (e.g., pinch, snuff, chew)<br>4 sb6___4 Biddis, kreteks<br>5 sb6___5 Hookahs, waterpipe or sheesha (with nicotine)<br>6 sb6___6 I wasn't using other forms of tobacco while vaping to quit<br><br>Custom alignment: LV<br>Field Annotation: @NONEOFTHEABOVE=6
107	sb10<br><br>Show the field ONLY if:<br>([smkstat]='1' or [smkstat] ='2') and [sb9]<>'1'	How likely are you to try vaping to quit during your next quit attempt?	radio<br>1 Very likely<br>2 Likely<br>3 Not likely<br>4 Very unlikely<br><br>Custom alignment: LV
108	sb11<br><br>Show the field ONLY if:<br>[smkstat]='3'	On a scale of 0 to 10, where 0 is not at all confident and 10 is extremely confident, how confident are you that you can stay off cigarettes for good?	slider<br>Slider labels: Not at all confident<br>0, 5, Extremely confident<br>10<br>Custom alignment: LH
109	behaviour_survey_complete	Section Header: *Form Status*<br><br>Complete?	dropdown<br>0 Incomplete<br>1 Unverified<br>2 Complete
	Instrument: **Nonvaper Survey** (nonvaper_survey)		Collapse
110	vq_intro2	The next questions ask about your vaping frequency, device, and behaviours while vaping to quit in the PAST 12 MONTHS. If you have tried vaping to quit more than once in the past year, please think about the time you LAST tried vaping to quit smoking.	descriptive
111	vbh7_nv	The LAST time you vaped to quit, approximately how many times a day did you vape?	radio<br>1 Less than daily<br>2 Once per day<br>3 2-4 times per day<br>4 5-9 times per day<br>5 10-14 times per day<br>6 15-19 times per day<br>7 20-29 times per day<br>8 30 or more times per day<br><br>Custom alignment: LV

112	vbh8_nv	While vaping to quit, approximately how many puffs did you take each time you vaped?	radio<br>1 Less than 5<br>2 5-9<br>3 10-14<br>4 15-19<br>5 20-29<br>6 30 or more<br><br>Custom alignment: LV
113	vbh9_nv	How soon after you woke up did you have your first vape of the day?	radio<br>1 0-5 minutes<br>2 6-15 minutes<br>3 16-30 minutes<br>4 31-60 minutes<br>5 61-120 minutes<br>6 More than 120 minutes<br><br>Custom alignment: LV
114	vbh22<br><br>Show the field ONLY if:<br>[vstat] = '4' or [vstat] = '5'	Why did you stop vaping? Check all that apply.	checkbox<br>1 vbh22___1 I didn't like the taste<br>2 vbh22___2 I didn't like the experience<br>3 vbh22___3 I don't like how the device looks<br>4 vbh22___4 It's too complicated to use<br>5 vbh22___5 It's too expensive<br>6 vbh22___6 It's too hard to get<br>7 vbh22___7 It made me feel sick<br>8 vbh22___8 I was still craving nicotine<br>9 vbh22___9 I was getting too much nicotine<br>10 vbh22___10 I was concerned about my health<br>11 vbh22___11 It didn't help me quit smoking cigarettes<br>12 vbh22___12 I quit smoking cigarettes and don't need to vape anymore<br>13 vbh22___13 I feel judged when vaping in public<br>14 vbh22___14 I can't use them in enough places<br>15 vbh22___15 Other (specify)<br><br>Custom alignment: LV
115	vbh22_other<br><br>Show the field ONLY if:<br>[vbh22(15)] = '1'	Please specify any other reason(s) you stopped vaping.	text<br>Custom alignment: LV
116	dev1_nv_photos	Section Header:<br>The next questions ask about your vaping devices and e-juice you used while vaping to try and quit smoking cigarettes.<br><br>Please use the letters above the images to answer the next question.	descriptive
117	vapeimages2		descriptive

118	dev1_nv	While vaping to quit in the PAST 12 months, what types of vaping devices did you use? Check all that apply.	checkbox<br>1 dev1_nv___1 A - A disposable cig-a-like vaping device that you can use one time or recharge the battery and use many times<br>2 dev1_nv___2 B - A simple pen-like device that has a battery and a tank that you refill with liquids<br>3 dev1_nv___3 C - An advanced box or tubular device that has a customized battery and tank you refill with liquids<br>4 dev1_nv___4 D - A pod system or pod vape that can be used one time with a prefilled pod, or they can be recharged with a USB charger and use pods or cartridges that insert in the device (e.g., JUUL, MYLE, MyBlu, Vype, Logic, Breeze 2, etc.)<br>5 dev1_nv___5 Other device (specify)<br>6 dev1_nv___6 Don't know<br><br>Custom alignment: LV<br>Field Annotation: @NONEOFTHEABOVE=8
119	dev1_nv_other<br><br>Show the field ONLY if:<br>[dev1_nv(5)] = '1'	Please specify the other type(s) of vaping device(s) you used while vaping to quit in the PAST 12 months.	text<br>Custom alignment: LV
120	brand_nv	What specific brand and model of vaping device did you use when you LAST vaped to try and quit smoking? (e.g., Aspire Breeze 2, Aspire Cobble, JUUL, Vype ePod, Smok Novo, VGOD Stig, GeekVape Aegis Legend, etc.)	text<br>Custom alignment: LV
121	dev2_nv	Was this vaping device disposable or rechargeable?	radio<br>1 Disposable<br>2 Rechargeable<br><br>Custom alignment: LV
122	dev3_nv	Did your device have a tank system or did it use cartridges?	radio<br>1 Tank system<br>2 Cartridges<br><br>Custom alignment: LV
123	dev4_nv<br><br>Show the field ONLY if:<br>[dev3_nv] = '1'	How many millilitres of e-juice did your tank system hold? If you do not know how many mL of e-juice your device held, please enter 999.	text (number)<br>Custom alignment: LV
124	dev5_nv<br><br>Show the field ONLY if:<br>[dev3_nv] = '1'	How often did you adjust the power settings (wattage, voltage, temperature) of the vaping device you used when you LAST vaped to quit?	radio<br>1 My device didn't have modifiable settings<br>2 I could modify the settings but I never changed them<br>3 I sometimes changed the settings<br>4 I often changed the settings<br>5 I always changed the settings<br><br>Custom alignment: LV
125	dev13_nv<br><br>Show the field ONLY if:<br>[dev3_nv] = '2'	Did you refill your pods/cartridges with e-juice?	yesno<br>1 Yes<br>0 No<br><br>Custom alignment: LV

<table>
<tr><td>126</td><td>dev6_nv

Show the field ONLY if:
[dev3_nv] = '1'</td><td>What was the voltage (v) of the device you LAST used when vaping to quit? Please answer in number of volts (e.g., 3.3). If you used a variable voltage device, what is the voltage you most often vaped at? Please only enter the number, not the words 'v' or 'volts'. If you do not know, please enter 999.</td><td>text (number)
Custom alignment: LV</td></tr>
<tr><td>127</td><td>dev7_nv

Show the field ONLY if:
[dev3_nv] = '1'</td><td>What power did you operate your device at when you LAST vaped to quit? Please answer in watts (w) (e.g.,20). If you used a variable setting device, how many watts did you most often vape at? Please only enter the number, not the words 'w' or 'watts'. If you do not know how many watts your device operated at, please enter 999.</td><td>text (number)
Custom alignment: LV</td></tr>
<tr><td>128</td><td>dev8_nv

Show the field ONLY if:
[dev3_nv] = '1'</td><td>What is the resistance setting you operated your device at when you LAST vaped to quit? Please answer in ohms ohms (?) (e.g., 1.5). If you have a variable setting device, how many ohms did you most often vape at? Please only enter the number, not the words '?' or 'ohms'. If you do not know how many ohms your device operated at, please enter 999.</td><td>text (number)
Custom alignment: LV</td></tr>
<tr><td>129</td><td>dev9_nv</td><td>What flavour(s) did you typically use while LAST vaping to quit? Check all that apply.</td><td>checkbox
<table>
<tr><td>1</td><td>dev9_nv___1</td><td>Fruit</td></tr>
<tr><td>2</td><td>dev9_nv___2</td><td>Candy</td></tr>
<tr><td>3</td><td>dev9_nv___3</td><td>Dessert</td></tr>
<tr><td>4</td><td>dev9_nv___4</td><td>Beverage - alcohol</td></tr>
<tr><td>5</td><td>dev9_nv___5</td><td>Beverage - non alcohol</td></tr>
<tr><td>6</td><td>dev9_nv___6</td><td>Mint/menthol</td></tr>
<tr><td>7</td><td>dev9_nv___7</td><td>Tobacco</td></tr>
<tr><td>8</td><td>dev9_nv___8</td><td>Food</td></tr>
<tr><td>9</td><td>dev9_nv___9</td><td>Other (specify)</td></tr>
<tr><td>10</td><td>dev9_nv___10</td><td>Don't know</td></tr>
</table>
Custom alignment: LV
Field Annotation: @NONEOFTHEABOVE=10</td></tr>
<tr><td>130</td><td>dev9_nv_other

Show the field ONLY if:
[dev9_nv(9)] = '1'</td><td>Please specify the other flavour(s) you typically used when you LAST vaped to quit smoking cigarettes.</td><td>text
Custom alignment: LV</td></tr>
<tr><td>131</td><td>dev10_nv</td><td>What nicotine concentration did you typically use when you LAST vaped to quit?</td><td>radio
<table>
<tr><td>1</td><td>0 mg/ml (no nicotine)</td></tr>
<tr><td>2</td><td>1 - 4 mg/ml (0.1-0.4%)</td></tr>
<tr><td>3</td><td>5 - 8 mg/ml (0.5-0.8%)</td></tr>
<tr><td>4</td><td>9 - 14 mg/ml (0.9-1.4%)</td></tr>
<tr><td>5</td><td>15 - 20 mg/ml (1.5-2.0%)</td></tr>
<tr><td>6</td><td>21 - 24 mg/ml (2.1-2.4%)</td></tr>
<tr><td>7</td><td>25 - 40 mg/ml (2.5%-3.4%)</td></tr>
<tr><td>8</td><td>41 - 59 mg/ml (3.5%-5%)</td></tr>
<tr><td>9</td><td>60 mg/ml or more (>5%)</td></tr>
<tr><td>10</td><td>Don't know</td></tr>
</table>
Custom alignment: LV</td></tr>
<tr><td>132</td><td>dev11_nv

Show the field ONLY if:
[dev10_nv]<>'1'</td><td>What type of nicotine did you use when you LAST vaped to quit smoking?</td><td>radio
<table>
<tr><td>1</td><td>Nicotine salts</td></tr>
<tr><td>2</td><td>Free-based nicotine</td></tr>
<tr><td>3</td><td>Don't know</td></tr>
</table>
Custom alignment: LV</td></tr>
</table>

133	dev12_nv<br><br>Show the field ONLY if:<br>[dev3_nv]='1' or [dev13_nv]='1'	Which of the following VG/PG (vegetable glycerin/propylene glycol) ratio did you typically use when you LAST vaped to try and quit smoking?	radio<br>1 100/0 VG/PG<br>2 80/20 VG/PG<br>3 75/25 VG/PG<br>4 70/30 VG/PG<br>5 60/40 VG/PG<br>6 50/50 VG/PG<br>7 30/70 VG/PG<br>8 0/100 VG/PG<br>9 Other (specify)<br>10 Don't know<br><br>Custom alignment: LV
134	dev12_nv_other<br><br>Show the field ONLY if:<br>[dev12_nv] = '9'	Please specify the VG/PG (vegetable glycerin/propylene glycol) ratio you used when you LAST vaped to try smoking.	text<br>Custom alignment: LV
135	nonvaper_survey_complete	Section Header: *Form Status*<br><br>Complete?	dropdown<br>0 Incomplete<br>1 Unverified<br>2 Complete
Instrument: **Vaper Survey** (vaper_survey)			Collapse
136	vbh_intro	These next questions ask about your CURRENT vaping behaviours.	descriptive
137	vbh17	Would you say that you are...?	radio<br>1 Very addicted to vaping<br>2 Somewhat addicted to vaping<br>3 Not at all addicted to vaping<br>4 I don't know<br><br>Custom alignment: LV
138	vbh18	Are you planning to quit vaping completely...?	radio<br>1 Within the next month<br>2 Within the next 6 months<br>3 Sometime in the future beyond 6 months<br>4 I am not planning to quit vaping<br><br>Custom alignment: LV
139	vbh19	Stealth vaping is vaping in a public place where it was not allowed and you attempt to hide your vaping. How often do you STEALTH vape?	radio<br>1 Never<br>2 Rarely<br>3 Sometimes<br>4 Very often<br>5 Always<br><br>Custom alignment: LV

140	vbh20<br><br>Show the field ONLY if:<br>[vbh19]>1	Where have you STEALTH vaped? Check all that apply.	checkbox<br>1 vbh20___1 At work<br>2 vbh20___2 At school<br>3 vbh20___3 In a restaurant<br>4 vbh20___4 At the movies<br>5 vbh20___5 In bars / nightclubs<br>6 vbh20___6 While shopping<br>7 vbh20___7 Airport and /or airplane<br>8 vbh20___8 Public transportation<br>9 vbh20___9 Other (specify)<br><br>Custom alignment: LV
141	vbh20_other<br><br>Show the field ONLY if:<br>[vbh20(9)] = '1'	Please specify where you have STEALTH vaped?	text<br>Custom alignment: LV
142	vbh10	On how many days, of the past 30 days, did you vape? Enter a number from 1-30.	text (number, Min: 0, Max: 30)<br>Custom alignment: LV
143	vbh7_v	On the days that you vape, approximately how many times a day do you vape?	radio<br>1 Less than daily<br>2 Once per day<br>3 2-4 times per day<br>4 5-9 times per day<br>5 10-14 times per day<br>6 15-19 times per day<br>7 20-29 times per day<br>8 30 or more times per day<br><br>Custom alignment: LV
144	vbh8_v	Approximately how many puffs do you take each time you vape?	radio<br>1 Less than 5<br>2 5-9<br>3 10-14<br>4 15-19<br>5 20-29<br>6 30 or more<br><br>Custom alignment: LV
145	vbh9_v	How soon after you wake up do you have your first vape of the day?	radio<br>1 0-5 minutes<br>2 6-15 minutes<br>3 16-30 minutes<br>4 31-60 minutes<br>5 61-120 minutes<br>6 More than 120 minutes<br><br>Custom alignment: LV
146	vbh11	Do you sometimes wake up at night to vape?	yesno<br>1 Yes<br>0 No<br><br>Custom alignment: LV

147	vbh12<br><br>Show the field ONLY if:<br>[vbh11]='1'	How many nights per week do you typically wake up to vape?	dropdown<br>0 0<br>1 1<br>2 2<br>3 3<br>4 4<br>5 5<br>6 6<br>7 7<br><br>Custom alignment: LV
148	vbh13	Do you ever have strong cravings to vape?	yesno<br>1 Yes<br>0 No<br><br>Custom alignment: LV
149	vbh14<br><br>Show the field ONLY if:<br>[vbh13]='1'	Over the past week, how strong have the urges to vape been?	radio<br>0 None<br>1 Slight<br>2 Moderate<br>3 Strong<br>4 Extremely strong<br><br>Custom alignment: LV
150	vbh15_intro	Please answer Yes or No for each of the following statements.	descriptive
151	vbh15_1	Do you vape because it is really hard to quit?	radio (Matrix)<br>1 Yes<br>0 No
152	vbh15_2	Do you find it hard to keep from vaping in places where you are not supposed to (e.g., movie theaters, libraries, etc.)?	radio (Matrix)<br>1 Yes<br>0 No
153	vbh15_3	Do you feel more irritable when you can't vape?	radio (Matrix)<br>1 Yes<br>0 No
154	vbh15_4	Do you feel nervous, restless, or anxious when you can't vape?	radio (Matrix)<br>1 Yes<br>0 No
155	device_intro	Section Header:<br>The next questions ask about your vaping device and e-juice.<br><br>Please use the letters above the images to answer the next question.	descriptive
156	vapeimages1		descriptive

157	dev1_v	What type of vaping device do you usually use?	radio<br>1 A - A disposable cig-a-like vaping device that you can use one time or recharge the battery and use many times<br>2 B - A simple pen-like device that has a battery and a tank that you refill with liquids<br>3 C - An advanced box or tubular device that has a customized battery and tank you refill with liquids<br>4 D - A pod system or pod vape that can be used one time with a prefilled pod or they can be recharged with a USB charger and use pods or cartridges that insert in the device (e.g., JUUL, MYLÉ, MyBlu, Vype, Logic, Breeze 2, etc.)<br>5 Other device (specify)<br>8 Don't know<br><br>Custom alignment: LV
158	dev1_v_other<br><br>Show the field ONLY if:<br>[dev1_v] = '5'	Please specify the type of vaping device you usually use.	text<br>Custom alignment: LV
159	brand_v	What specific brand and model of vaping device do you use? (e.g., Aspire Breeze 2, Aspire Cobble, JUUL, Vype ePod, Smok Novo, VGOD Stig, GeekVape Aegis Legend, etc.)	text<br>Custom alignment: LV
160	dev4_v<br><br>Show the field ONLY if:<br>[dev1_v] = '2' or [dev1_v] = '3'	How many millilitres of e-juice does your tank system hold? If you do not know how many mL of e-juice your device held, please enter 999.	text (number)<br>Custom alignment: LV
161	dev5_v<br><br>Show the field ONLY if:<br>[dev1_v] = '3'	How often do you adjust the power settings (wattage, voltage, temperature) on your device?	radio<br>1 My device doesn't have modifiable settings<br>2 I can modify the settings but I never change them<br>3 I sometimes change the settings<br>4 I often change the settings<br>5 I always change the settings<br><br>Custom alignment: LV
162	dev13_v<br><br>Show the field ONLY if:<br>[dev1_v] = '4'	Do you refill your pods/cartridges with e-juice?	yesno<br>1 Yes<br>0 No<br><br>Custom alignment: LV
163	dev6_v<br><br>Show the field ONLY if:<br>[dev1_v] = '3'	What is the voltage (v) of your device? Please answer in number of volts (e.g., 3.3). If you used a variable voltage device, what is the voltage you most often vape at? Please only enter the number, not the words 'v' or 'volts'. If you do not know, please enter 999.	text (number)<br>Custom alignment: LV
164	dev7_v<br><br>Show the field ONLY if:<br>[dev1_v] = '3'	What power do you operate your device at? Please answer in watts (w) (e.g.,20). If you used a variable setting device, how many watts do you most often vape at? Please only enter the number, not the words 'w' or 'watts'. If you do not know, please enter 999.	text (number)<br>Custom alignment: LV
165	dev8_v<br><br>Show the field ONLY if:<br>[dev1_v] = '3'	What is the resistance setting you operate your device at? Please answer in ohms (?) (e.g., 1.5). If you have a variable setting device, how many ohms do you most often vape at? Please only enter the number, not the words 'Ω' or 'ohms'. If you do not know, please enter 999.	text (number)<br>Custom alignment: LV

166	dev9_v	What flavour(s) do you usually use when you vape? Check all that apply.	checkbox<br>1 dev9_v___1 Fruit<br>2 dev9_v___2 Candy<br>3 dev9_v___3 Dessert<br>4 dev9_v___4 Beverage - alcohol<br>5 dev9_v___5 Beverage - non alcohol<br>6 dev9_v___6 Mint/menthol<br>7 dev9_v___7 Tobacco<br>8 dev9_v___8 Food<br>9 dev9_v___9 Other (specify)<br>10 dev9_v___10 Don't know<br><br>Custom alignment: LV<br>Field Annotation: @NONEOFTHEABOVE=10
167	dev9_v_other<br>Show the field ONLY if:<br>[dev9_v(9)] = '1'	Please specify the other flavour(s) you typically use.	text<br>Custom alignment: LV
168	dev10_v	What nicotine concentration do you typically use?	radio<br>1 0mg/ml (no nicotine)<br>2 1 - 4 mg/ml (0.1-0.4%)<br>3 5 - 8 mg/ml (0.5-0.8%)<br>4 9 - 14 mg/ml (0.9-1.4%)<br>5 15 - 20 mg/ml (1.5-2.0%)<br>6 21 - 24 mg/ml (2.1-2.4%)<br>7 25 - 40 mg/ml (2.5%-3.4%)<br>8 41 - 59 mg/ml (3.5%-5%)<br>9 60 mg/ml or more (>5%)<br>10 Don't know<br><br>Custom alignment: LV
169	dev11_v<br>Show the field ONLY if:<br>[dev10_v]<>'1'	What type of nicotine do you use?	radio<br>1 Nicotine salts<br>2 Free-based nicotine<br>3 Don't know<br><br>Custom alignment: LV
170	vbh21	During the past 12 months, how has the nicotine concentration of your e-juice/pods/cartridges changed?	radio<br>1 It has decreased<br>2 It has stayed the same<br>3 It has increased<br>4 I don't know<br><br>Custom alignment: LV

171	dev12_v<br>Show the field ONLY if:<br>([dev1_v]='2' or [dev1_v]='3') or [dev13_v]='1'	Which of the following VG/PG (vegetable glycerin/propylene glycol) ratios do you usually use?	radio<br>1 100/0 VG/PG<br>2 80/20 VG/PG<br>3 75/25 VG/PG<br>4 70/30 VG/PG<br>5 60/40 VG/PG<br>6 50/50 VG/PG<br>7 30/70 VG/PG<br>8 0/100 VG/PG<br>9 Other (specify)<br>10 Don't know<br>Custom alignment: LV
172	dev12_v_other<br>Show the field ONLY if:<br>[dev12_v] = '9'	Please specify the VG/PG (vegetable glycerin/propylene glycol) ratio you usually use.	text<br>Custom alignment: LV
173	vaper_survey_complete	Section Header: *Form Status*<br>Complete?	dropdown<br>0 Incomplete<br>1 Unverified<br>2 Complete
Instrument: **Demo** (demo)			Collapse
174	demo_intro	You're almost done! The final questions are about you. This information is for classification purposes. Your responses are confidential.	descriptive
175	sb14_intro	How frequently do you currently use each of the following?	descriptive
176	sb14a	Marijuana (a joint, pot, weed, cannabis, hash or hash oil)	radio (Matrix)<br>1 Daily or almost daily<br>2 Occasionally<br>3 Not at all<br>4 I have NEVER used this product
177	sb14b	Alcohol	radio (Matrix)<br>1 Daily or almost daily<br>2 Occasionally<br>3 Not at all<br>4 I have NEVER used this product
178	sb14c	Hookah, shisha or waterpipe	radio (Matrix)<br>1 Daily or almost daily<br>2 Occasionally<br>3 Not at all<br>4 I have NEVER used this product
179	sb14d	Other tobacco products such as cigars, pipes, chewing tobacco, bidis, kreteks	radio (Matrix)<br>1 Daily or almost daily<br>2 Occasionally<br>3 Not at all<br>4 I have NEVER used this product

180	sb14e	Heated tobacco which uses tobacco leaf (e.g., IQOS, glo, Ploom Tech)	radio (Matrix)<br>1 Daily or almost daily<br>2 Occasionally<br>3 Not at all<br>4 I have NEVER used this product
181	age	How old are you?	text (number, Min: 18, Max: 100)<br>Custom alignment: LV
182	gender	How do you describe yourself?	radio<br>1 Male<br>2 Female<br>3 Transgender<br>4 None of the above<br>5 Prefer not to answer<br>Custom alignment: LV
183	edu	What is the highest level of education you have completed?	radio<br>1 Some elementary or high school<br>2 Completed high school<br>3 College diploma<br>4 University or post graduate degree<br>Custom alignment: LV
184	employ	How would you best describe your current employment status?	radio<br>1 Student<br>2 Full-time paid employment<br>3 Part-time paid employment<br>4 Self-employed<br>5 Unemployed<br>6 Working without pay<br>7 Other (please specify)<br>Custom alignment: LV
185	employ_other<br>Show the field ONLY if:<br>[employ] = '7'	Please specify your current employment status.	text<br>Custom alignment: LV
186	married	What is your marital status?	radio<br>1 Single<br>2 Married or living with a partner<br>3 Divorced/Separated<br>4 Widowed<br>Custom alignment: LV

187	race	How would you BEST describe your racial identity? Check all that apply.	checkbox<br>1 race___1 White<br>2 race___2 Asian<br>3 race___3 Black<br>4 race___4 Indigenous<br>5 race___5 Middle Eastern<br>6 race___6 Latin American<br>7 race___7 Other (specify)<br>8 race___8 Prefer not to answer<br><br>Custom alignment: LV<br>Field Annotation: @NONEOFTHEABOVE=8
188	race_other<br><br>Show the field ONLY if:<br>[race(7)] = '1'	Please specify how you would BEST describe your racial identity.	text<br>Custom alignment: LV
189	fup_consent	Would you be interested in being contacted for further research on vaping to quit smoking?	radio<br>1 Yes<br>0 No<br><br>Custom alignment: LV
190	pt_experience	In the space below, please add any other details or information you would like to share about your experience while while vaping to quit smoking cigarettes.	notes<br>Custom alignment: LV
191	demo_complete	Section Header: *Form Status*<br>Complete?	dropdown<br>0 Incomplete<br>1 Unverified<br>2 Complete

Instrument: **Close** (close) Collapse

192	close	Thank you!<br><br>You will receive a $10 e-gift card from Giftbit within 10 to 15 business days - follow the instructions in the Giftbit email to claim your gift. Your name will also be entered into a draw for one $500 VISA gift card, or one of two $250 VISA gift cards. Draws will be held on or before September 30, 2019.<br><br>For more information about our study, please email the study coordinator.<br><br>Please click the "Submit" button below. Have a great day!	descriptive
193	close_complete	Section Header: *Form Status*<br>Complete?	dropdown<br>0 Incomplete<br>1 Unverified<br>2 Complete
